# Development and validation of search hedges for Transgender and Gender Diverse (TGD) populations in Ovid MEDLINE and Ovid APA PsycInfo

**DOI:** 10.1371/journal.pone.0351303

**Published:** 2026-06-12

**Authors:** Ryn Gagen, Scott Marsalis, Chi Dinh, Milo Applejohn, Rosa Shah, Ophelia Xiner Tong, Zack Marshall, Avery Everhart

**Affiliations:** 1 University Libraries, University of Minnesota, Minneapolis, Minnesota, United States of America; 2 Department of Community Health Sciences, Cumming School of Medicine, University of Calgary, Calgary, Alberta, Canada; 3 Faculty of Health Sciences, Simon Fraser University, Burnaby, British Columbia, Canada; 4 Department of Geography, School of Arts, The University of British Columbia, Vancouver, British Columbia, Canada; 5 O’Brien Institute for Public Health, University of Calgary, Calgary, Alberta, Canada; 6 School of Social Work, McGill University, Montreal, Quebec, Canada; 7 Center for Applied Transgender Studies, Chicago, Illinois, United States of America; Mount Sinai School of Medicine: Icahn School of Medicine at Mount Sinai, UNITED STATES OF AMERICA

## Abstract

**Introduction:**

This paper describes the development and validation of highly sensitive search hedges for Ovid MEDLINE and Ovid APA PsycInfo that effectively identify literature on transgender and gender diverse (TGD) populations.

**Methods:**

Two librarians developed the search hedges using relevant keywords and controlled vocabulary terms, building on previous work on identifying transgender populations in evidence synthesis. The hedges were tested and refined to capture diverse and expansive gender identities across cultures and disciplines. The hedges were validated for sensitivity using a gold standard set of 144 articles from the Knowsy portal of evidence syntheses tagged as Two-Spirit, transgender, or gender non-binary. To assess precision an international research team of subject experts independently screened a randomized sample of search results in a two-stage screening process with an additional screener resolving disputes.

**Results:**

The final search hedges demonstrated 100% sensitivity in both MEDLINE and APA PsycInfo, identifying all 144 relevant articles from the Knowsy gold standard set. The MEDLINE search hedge achieved a 71% precision, and the APA PsycInfo hedge achieved a 67% precision. These results balance comprehensive retrieval while minimizing non-relevant articles for an efficient screening process.

**Conclusions:**

These search hedges in MEDLINE and APA PsycInfo are valuable tools for researchers and librarians to more effectively identify literature on TGD populations. These tools will be crucial for ongoing work in addressing gaps in research and health disparities faced by TGD populations and will be particularly valuable for researchers conducting evidence synthesis projects related to this population.

## Introduction

The number of academic articles being published has grown tremendously over the past decade, with the number of scientific articles published increasing 47% between 2016 and 2022 [1]. Information scientists, librarians, researchers, clinicians, and students now face the challenge of navigating these ever-growing bodies of work to find relevant information in a timely manner. Librarians and other information professionals supporting evidence syntheses have developed multiple strategies to address this challenge, one of which is the use of search hedges.

Search hedges, also called search filters, are tools that aid in the effective and efficient retrieval of literature on a topic. Search hedges are carefully constructed combinations of search strings that researchers and information professionals can utilize, as pre-constructed components of a larger, comprehensive search strategy in evidence synthesis projects, to retrieve records in a database relating to a facet of their research question — including specific populations, methodologies, diagnoses, or geographic areas [2–5]. In particular, search hedges can improve the comprehensiveness and sensitivity of database queries when searching for complex concepts and populations that have significant language variability. Researchers can incorporate search hedges as an integral part of their approach to evidence synthesis, thus making their searches more complete and reducing the risk of missing relevant literature.

Hedges typically incorporate terms from a database’s official thesaurus, such as Medical Subject Headings (MeSH), which are the controlled vocabulary terms used in the MEDLINE database. Hedges also include additional free text terms likely to be included elsewhere in a record’s searchable metadata fields, such as the title, abstract, or author-supplied keywords. Hedges may be developed by a librarian based on their knowledge of a database’s thesaurus, record structure, content, and interface, in combination with researcher or clinician subject knowledge. Ideally, search hedges are validated by testing their sensitivity [6,7], also referred to as recall, against a “gold standard” set of known relevant records. Gold standard sets may be generated by hand searching relevant journals, through relative recall, or by testing against an existing gold standard set derived from multiple bibliographic databases and other sources [5]. They are also evaluated based on their precision, which is the ratio of relevant to irrelevant records retrieved [8]. A challenge of search hedge development is balancing sensitivity and precision, as they are inversely related [5]. Reviews on topics with developing or dynamic terminology require a more sensitive strategy. When utilizing a search hedge, information professionals and researchers should consider if the hedge has been validated, if it optimizes sensitivity or precision, when it was last updated, and whether it needs to be adapted to suit their specific research question. While a more thorough explanation of search hedges as tools for more general use is beyond the scope of this study, others have described the landscape of existing topic search hedges [4]. There are a variety of sources for validated search hedges. For example, the InterTASC Information Specialists’ Sub-Group Search Filter Resource provides access to published and unpublished search hedges as well as information about how to critically develop and critically appraise search hedges [9].

Developing a search hedge for Transgender and Gender Diverse (TGD) populations represents an opportunity to address many of the challenges in constructing, validating, and applying hedges for literature reviews and evidence syntheses that we outline above. Searching for literature related to Transgender and Gender Diverse (TGD) populations is a persistent challenge within major academic databases. Existing search strategies often fail to capture the full range of gender diversity — particularly when it comes to culturally specific gender identities, Indigenous systems of sex and gender, and evolving gender terminology. Indeed, Indigenous knowledge organization scholars have suggested that two-spirit, both as a broad category and the nation-specific identities that may fall under the larger heading, poses challenges to traditional archiving, information recall and knowledge organization systems [10,11]. To that end, the authors are unaware of any existing research on the discoverability of culturally specific gender identities within scholarly databases. Additionally, database indexing and common writing practices, such as authors using the full 2SLGBTQIA+ acronym when only a subset of this population is included in a study, can conflate gender identity with sexual orientation or omit TGD populations altogether, leading to incomplete or skewed search results. Given the barriers to identifying literature relevant to TGD populations, there is a need for validated, adaptable search hedges that reliably retrieve relevant literature across disciplines and database platforms.

The search strategies presented here have been carefully cultivated to balance sensitivity with precision. Terms that produced excessive noise (irrelevant search results) have been selectively pruned to improve relevance, while other terms that do not currently retrieve many records have been deliberately included for future growth as scholarly research adapts to emerging terminology. Developed collaboratively by librarians embedded in both medical and social science disciplines, along with subject matter experts, we present hedges for two of the predominant databases indexing literature relating to the physical and mental health of humans, MEDLINE, which is the United States National Library of Medicine’s premier database for the health sciences containing more than 31 million bibliographic records [12] and APA PsycInfo, the American Psychological Association’s premier database of behavioral and social sciences containing over 5 million bibliographic records [13]. We chose to develop hedges via the Ovid platform for both databases because of its advanced search functionality, which include adjacency operators, advanced wildcard functions, built-in search filters and advanced limits. Thus, when we refer to the hedges throughout we specify that they are designed for use with Ovid MEDLINE and Ovid APA PsycInfo. Rather than serving as static tools, these ‘living’ search hedges are designed to be regularly updated to capture new and relevant terminology. Through active maintenance and user adaptation, these hedges should be refined and expanded to reflect changes in language, indexing preferences, and the needs of researchers working with TGD populations over time.

The TGD search hedges presented here contribute to ongoing work in developing and validating PubMed search hedges for 2SLGBTQIA+ populations broadly [14]. Search hedges and resource guides have also been developed specifically for finding literature on TGD populations, but none of these strategies have been validated [15–17]. These hedges aim to contribute to this literature by providing the first validated search hedges for TGD populations for Ovid MEDLINE and Ovid APA PsycInfo. Although these hedges are designed and validated on the Ovid platform, they are constructed in such a way to facilitate translation to other platforms, including PubMed, EBSCOhost, ProQuest, and APA PsycNet.

## Methods

The validation process of these two search hedges was previously described in a published study protocol [18]. Here, we describe the iterative development of the hedges — including the initial selection, management, and structure of search terms; adjustments to the search hedges after the preliminary validation process; and finally, the results of the full validation based on random samples from the results of each respective database. The full search strategies for the Ovid MEDLINE and Ovid APA PsycInfo search hedges can be found under supplemental files at the end of this paper, including notes where updates have been made since the validation process to incorporate controlled vocabulary changes in database thesauri.

### Search term selection

Selection of search terms drew upon professional subject expertise within the author team, personal lived experience of authors who are TGD themselves, and several existing resources. To create comprehensive search strategies, the terminology utilized in the search hedges intentionally transcend narrow definitions of TGD populations in medical contexts. The search hedges include culturally specific gender identity terms adapted from search strategies used in the transgender health guideline commissioned by the World Health Organization [19], the Trans Evidence Map [20], and a map of global gender-diverse identities published by Independent Lens [21]. Lastly, we consulted the initial searches used to populate Knowsy [22], a comprehensive online portal for 2SLGBTQIA+ evidence syntheses, which, while broader in scope, provided some additional terms. While no limits such as language, geography, and methodology were applied to our selection of search terms, inherent biases may be present in the terminology of these search hedges given their development by anglophones for use in English language databases. Culturally specific terms, such as “fa’afafine” or “muxe,” may be used out of context in ways that do not necessarily align with how they are used in cultural contexts, or in ways that subsume autochthonous understandings of gender under the heading of transgender or gender diverse.

While gender-affirming care is associated with TGD people, many gender-affirming interventions were developed for and are sought out by cisgender people, that is non-transgender individuals whose current gender identities correspond to the sexes they were assigned at birth. Therefore, the search hedges are intentionally selective in the inclusion of gender-affirming care terminology. The included terminology related to medical transition reflect common types of surgeries, procedures, and interventions primarily utilized for transgender and gender diverse populations — such as facial feminization, metoidioplasty, neovagina, and voice masculinization. Terminology like “hormone replacement therapy” and the names of individual hormones, medications, and surgeries that are used in both trans- and cisgender populations were excluded. Another example of medical transition terminology intentionally excluded from the search hedges are classes of drugs commonly used in gender-affirming care regimens, such as GnRH agonists. GnRH agonists can be prescribed as ‘puberty blockers’ for both transgender youth and for cisgender youth diagnosed with precocious puberty [23]. Specific medication names, such as cyproterone acetate (brand name Androcur), which is typically used for prostate cancer treatment as well as an anti-androgen in a gender-affirming hormone therapy regimen, were also excluded [24].

### Data management process

To ensure transparency and reproducibility in the search hedge development process, all search terms were systematically collated and organized within a collaboratively maintained spreadsheet that was discussed between the two librarians at regular meetings. Organizing terms within the spreadsheet allowed for the tracking and testing of synonyms and word variants to maximize retrieval scope. Each librarian made notes on the spreadsheet to iteratively refine terminology to include in the hedges based on their performance in each database. Database-specific controlled vocabulary terms and free text keywords were separated within the spreadsheet to assess their relative performance, and the librarians ensured that any controlled vocabulary terms were reflected in corresponding free text terms to account for inconsistencies in database indexing. Search terms were also sorted into thematic clusters to ensure comprehensive representation of several different facets of TGD research. In addition to organizing terms, the spreadsheet facilitated conversations with the entire research team when it came to larger decisions on scope and sensitivity. When the team reached a consensus on whether to remove or retain individual terms in the search hedges, the spreadsheet assisted with documenting the decision-making process.

### Search hedge structure

The search hedges were developed in the Ovid platform for two predominant academic databases, Ovid MEDLINE and Ovid APA PsycInfo. This decision reflects our determination that these are predominant databases in the health sciences and social sciences where there is a need for effective retrieval of TGD literature for research. Search hedges can be translated across different databases and between different interfaces and platforms that search the same database. Glanville et al. (2019) [25] provide a case study detailing how to translate a validated search hedge from one platform to another. For ease of translation across different databases, the controlled vocabulary terms for each search hedge have been grouped in the first line of each respective hedge. These database-specific controlled vocabulary terms are the MeSH terms for Ovid MEDLINE and the APA Thesaurus of Psychological Index terms for Ovid APA PsycInfo. The other database-specific elements that would need to be translated for other databases or platforms would be the field codes (such as “.ti,ab,kf,kw,ot,oa,cl.” or “.tw.”) and the adjacency operator (“adj5”) that we tried to minimize. The human limiter for Ovid MEDLINE (line 12) is specific to that database to mitigate indexing errors. We tried to limit the need to translate other database-specific syntax such as advanced wildcard operators to reduce potential errors in translation.

Since access to specific databases and database platforms may vary depending on institutional affiliation and subscriptions, database operators such as advanced wildcards and adjacency were intentionally avoided or minimized to facilitate translation to other database platforms. Because of the prominence and accessibility of PubMed in health sciences research, the MEDLINE hedge was developed in the Ovid MEDLINE® ALL < 1946-Present> database multi-segment, which is identical in coverage to PubMed [26]. The Ovid MEDLINE search hedge utilizes the field codes “.ti,ab,kf,kw,ot,oa,cl,” which searches the title, abstract, author keywords, other titles, other abstracts, and collection titles of a record [27]. These field codes were intentionally selected to match PubMed’s [tiab] field code. For the Ovid APA PsycInfo hedge, the “.tw” field code was selected due to its close alignment with [tiab] in PubMed [28,29]. We chose field codes that align with [tiab] in PubMed in order to capture search terms in the title, abstract, or keywords fields of search results while still reducing irrelevant results from additional metadata fields. Neither Ovid MEDLINE nor Ovid APA PsycInfo allow searching ful text and other metadata fields (e.g., journal title and cited references) typically introduce unwanted or irrelevant results. Searching title, abstract, controlled vocabulary terms, and author-provided keywords is standard practice in evidence synthesis. Despite efforts to avoid using database syntax operators that may be difficult to translate to other databases, line 10 in each search hedge was developed in February 2024 after pilot validation to further reduce noise related to the keyword phrase “gender neutral.” This line was kept separate in each hedge to ease translation to databases that do not have adjacency operators.

The search hedges were designed in structured blocks to enhance adaptability for specific research questions. This approach allows for more modularity, making it easier to refine and adapt individual components of the hedges without disrupting the entire search strategy. For example, when MeSH headings relating to gender-affirming procedures were updated in 2025, it was easy to update the validated hedge [30]. By isolating different elements of transgender and gender diverse terminology in modular blocks, users can make more intentional choices to tailor searches for different goals, research questions, contexts, or databases. This method aligns well with evidence synthesis principles, where structured reproducible searches that are well-balanced between sensitivity and specificity are essential. After iterative testing, these search hedges were developed to have nine independent blocks (Table 1). Some of the blocks have a functional component for ease of translation across multiple databases, such as the separation of controlled vocabulary terms from other free-text keywords. However, most of the blocks are organized around a semantic component that researchers may want to narrow down a search on, such as transmasculine or transfeminine identities, culturally specific gender identity terms, or keywords related to gender-affirming care and medical transition. Terms are alphabetized within each block to facilitate ease of navigation and review for errors in translation. Table 1 illustrates the block structure utilized across both hedges, using the Ovid MEDLINE hedge as validated as an example.

**Table 1:** Modular block structure of the search hedges.

Block category	Description	Ovid MEDLINE hedge (June 2024)
**Controlled vocabulary terms**	Controlled vocabulary terms (MeSH terms, APA Thesaurus of Psychological Index terms).	exp Gender Dysphoria/ or exp Health Services for Transgender Persons/ or exp Sex Reassignment Procedures/ or exp Sex Reassignment Surgery/ or exp “Sexual and Gender Disorders”/ or exp Transgender Persons/ or exp Transsexualism/ or exp Transvestism/
**Transgender**	Terms related to transgender people in general.	(“trans bod*” or “trans elder*” or “trans experienc*” or “trans folk*” or “trans folx*” or “trans gender*” or “trans ident*” or “trans individual*” or “trans parent*” or “trans people*” or “trans person*” or “trans selv*” or “trans senior*” or “trans sex*” or “trans spectrum” or “trans visib*” or “trans youth*” or transex* or transfolk* or transfolx* or transgender* or transident* or transpeople* or transperson* or transsex* or transvesti* or transvisib*).ti,ab,kf,kw,ot,oa,cl.
**Transmasculine**	Terms related specifically to transmasculine identities.	(AFAB or “assigned female” or “trans boy*” or “trans father*” or “trans guy*” or “trans male*” or “trans man” or “trans masc*” or “trans men” or transboy* or transguy* or transmale* or transman or transmasc* or transmen).ti,ab,kf,kw,ot,oa,cl.
**Transfeminine**	Terms related specifically to transfeminine identities.	(AMAB or “assigned male” or “trans female” or “trans femin*” or “trans femme*” or “trans girl*” or “trans mother*” or “trans woman” or “trans women” or transfemal* or transfemin* or transfemme* or transgirl* or transwoman or transwomen).ti,ab,kf,kw,ot,oa,cl.
**Gender identity**	Terms related to broader gender identities.	(“2 spirit*” or agender* or ((androgynous or androgyny) not bem*) or “atypical gender” or “bi gender*” or bigender* or “demi boy*” or “demi girl*” or demiboy* or demigirl* or “dissident gender” or “gender atypical*” or “gender bend*” or “gender binar*” or “gender creativ*” or “gender divers*” or “gender expans*” or “gender expression*” or “gender fluid*” or “gender flux*” or “gender identit*” or “gender inclusiv*” or “gender minorit*” or “gender modalit*” or “gender non conform*” or “gender nonconform*” or “gender queer*” or “gender questioning” or “gender varian*” or genderdivers* or genderexpans* or genderflu* or genderqueer* or genderquestioning or “minority gender*” or neutrois or “non binary*” or “non cisgender*” or nonbinar* or noncisgender* or (TGD not yangtze) or thirdgender* or “third gender*” or “third sex” or “third spirit*” or trigender or “two spirit*” or twospirit*).ti,ab,kf,kw,ot,oa,cl.
**Culturally specific terms**	Gender identities related to culturally specific third genders and roles.	((acault and myanmar) or achout or aikane or “akava ine” or “akava’ine” or alyha or aravani or aravanis or ashtime or bakla or bantut or basivi or berdache* or bissu or “brother boys” or brotherboys or burrnesha or calabai or calalai or dilbaa or “fa afafine” or “fa’afafine” or fakafefine or fakafifine or fakaleiti or femminiell* or guevedoche or hijra* or hirja* or hwame or irahuhua or irawhiti or kathoe* or kathoey or kathoy or katoey or khanith or “khwaja saraa” or “khwaja sira” or kocek or kothi or koti or ladyboy* or leiti or lhamana or machi or mahu or mahuvahine or mahuwahine or “mak nyah” or maknyah or mashoga or ((meti or metis) and Nepal) or muxe or muxes or muxhe or nadleehi or ninauposkitzipxpe or paknyah or palopa or panthi or “phuying kham phet” or pinapinaaine or quariwarmi or “sao praphet song” or sekrata or selrata or “sister girls” or sistergirls or skoptsy or “sworn virgin” or “sworn virgins” or tahine or takataapui or “tangata ira tane” or “tangata ira wahine” or transpinay* or transpinoy or travesti* or vakasalewalewa or wakatane or waria or whakawahine or winkte or xanith).ti,ab,kf,kw,ot,oa,cl.
**Gender** **-affirming care**	Terms related to medical transition and gender-affirming care.	(autogynephil* or “cross gender*” or “cross sex hormon*” or crossgender* or “gender adjustment” or “gender affirm*” or “gender chang*” or “gender confirm*” or “gender disorder*” or “gender dysphor*” or “gender euphor*” or “gender identity disorder*” or “gender incongruen*” or “gender re-assign*” or “gender reassign*” or “gender transition*” or “sex affirm*” or “sex chang*” or “sex re-assign*” or “sex reassign*” or “sex transition*” or “sexual dysphor*” or “sexual reassignment” or “trans affirm*” or transaffirm* or “facial feminis*” or “facial feminiz*” or “facial masculinis*” or “facial masculiniz*” or “genital reconstruct*” or genitoplasty or metoidioplasty or neophallus or neovagina or phalloplasty or “pregnant man” or “pregnant men” or “puberty block*” or “puberty suppress*” or vaginoplasty or “vocal feminis*” or “vocal feminiz*” or “vocal masculinis*” or “vocal masculiniz*”).ti,ab,kf,kw,ot,oa,cl.
**Drag and crossdressing**	Terms related to drag and crossdressing.	(“cross dress*” or crossdress* or “drag king*” or “drag queen*” or “female impersonat*” or “male impersonat*”).ti,ab,kf,kw,ot,oa,cl.
**Discrimination**	Terms related to discrimination against transgender individuals.	(misgender* or tranny or transphobi*).ti,ab,kf,kw,ot,oa,cl.
**Gender neutral**	Refined search string using adjacency to reduce irrelevant results.	(gender neutral adj5 (pronoun* or language or bathroom*)).ti,ab,kf,kw,ot,oa,cl.

Initial testing of the Ovid MEDLINE search hedge uncovered problematic indexing inconsistencies in the search results regarding the medical subject heading (MeSH) for “Humans.” Filtering search results utilizing the “Humans” MeSH term was important for the functionality of the Ovid MEDLINE search hedge due to the common phenomenon of sex change present across other species. In the MeSH thesaurus, “Humans” is a more specific subject heading under the broader “Animals” MeSH term, and there are studies within MEDLINE indexed with both the “Animals” and “Humans” subject headings to indicate mixed human and non-human studies. The recommended approach from the *Cochrane Handbook for Systematic Reviews of Interventions* to limit MEDLINE search results to human studies is to append the search string “NOT (animals NOT humans)” to identify and remove animal-only studies and exclude them from the search results using a double negative. Compared to human filters that may be built into databases, this strategy is a more sensitive way to limit searches to human studies only [31]. However, the utilization of even this sensitive approach recommended by Cochrane unintentionally excluded a significant number of relevant studies where human-only studies were incorrectly indexed as “Animals” instead of “Humans.” Thus, modifications in line 12 of the Ovid MEDLINE search hedge were made to identify and remove results indexed under specific portions of the “Animals” MeSH tree structure to avoid erroneously excluding human studies that were incorrectly indexed with only the “Animals” MeSH term.

While the need for a more complex human limiter was discovered before pilot screening and validation of the search hedges began, some additional changes to the search hedges were made based on irrelevant articles that appeared frequently in our pilot validation process. The term “eunuch,” which has historically been used as a gender identity term [32], only brought in irrelevant results, so the decision was made to remove the term entirely. The acronym TGD (transgender and gender diverse) also pulled in irrelevant results discussing the Three Gorges Dam of the Yangtze River Estuary, so the term was adapted to exclude “Yangtze” from those results (line 5 of the Ovid MEDLINE hedge, line 6 of the Ovid APA PsycInfo hedge). The final adaptation made to reduce significant sources of noise identified in pilot validation was the introduction of a proximity operator (ADJ on the Ovid platform) to the term “gender neutral” to only include results where “gender neutral” was used within five words of keywords related to pronouns, language, or bathrooms. While the hedges were developed with the intention to avoid operators that are not fully available in other frequently used databases like PubMed, this decision was made to reduce the number of irrelevant articles that were found describing concepts such as gender-neutral HPV vaccinations.

### Validation

As discussed in the study protocol [18], the validation process for the search hedges involved randomly sampling from the search results our hedges produced in Ovid MEDLINE and Ovid APA PsycInfo. The results from each database were uploaded to the web-based review management software EPPI-Reviewer [33] for screening. De-duplication between databases was not performed to preserve the distinctive sample for each database. These randomized samples from each database were screened to validate the sensitivity and precision of the search hedges.

The precision validation process utilized the two-stage screening model of title and abstract review followed by full text review. During the title and abstract screening phase, we categorized articles using the following five categories: 1) Exclude, 2) No_Abstract, 3) Mixed_Topics, 4) LGB_maybe_T, and 5) Include. Articles were excluded if they exclusively used binary gender language without clarification of the inclusion of transgender people (e.g., women and men, female and male), if they focused exclusively on intersex people and people with differences in sexual development, or if they were focused on the gender identities of cisgender people. Articles were included if they discussed TGD people or TGD-related topics. More specifically, records were included if they had disaggregated data reflecting inclusion of TGD participants in the case of empirical studies, and non-empirical records were included if they distinguished a substantive discussion of TGD people in specific ways rather than strictly as part of a larger umbrella category (such as LGBT). Articles that were categorized as exclude or include at title and abstract screening did not undergo full text screening because they had clearly demonstrated their relevance or lack of relevance to TGD people and topics. There were three additional categories used in title and abstract screening that determined which articles would be considered for the full text screening phase. The No_Abstract category was used for articles without abstracts that needed to be reviewed in further depth to address how substantive their engagement with TGD populations was in the full text. The category Mixed_Topics was applied to articles that were unclear whether or not TGD people would be mentioned. The LGB_maybe_T category was used for articles that used some variation of an umbrella 2SLGBTQIA+ acronym for their study population, yet it was unclear if there was a significant indication of transgender study subjects or discussions applicable to TGD individuals.

During the full text screening phase, the only two categories available for screeners to choose from were Include or Exclude. Articles were excluded if they did not substantively engage with TGD individuals or related topics. This means that if the word “transgender” was in the abstract of an article through authors spelling out an acronym like 2SLGBTQIA+ without significant discussion of transgender or gender diverse individuals specifically, those articles were excluded. On the other hand, articles were included if they substantially engaged with TGD populations, were directly relevant to TGD topics, or if participant data was reported separately for TGD study subjects.

While the validation process has been described in detail in the published protocol [18], it is worth noting that the screening process involved a core team of four reviewers who independently reviewed randomized sets from within the random samples for each database. For both title and abstract and full text screening phases, articles were independently screened by at least two trained reviewers following the established inclusion and exclusion criteria for each category. Those four reviewers were overseen by two other team members responsible for the study design. In the case of disagreements across reviewers on individual records, consensus was reached among the study team to reconcile disagreements and determine which of the five categories a title and abstract record belonged to, or, in the final phase, whether to include or exclude a record based on a full text review. We discuss the consensus process more fully in the published protocol [18], and in the present publication we describe the results of that process, including agreement rates across the review team.

### Search term insights

The development and validation process uncovered search terms with unclear relevance to TGD topics, prompting a more detailed examination of the context in which these terms appeared and whether these unclear terms appeared alongside other TGD-related terms. These terms with unclear relevance included: “gender inclusive” or “gender inclusivity,” “gender diverse” or “gender diversity,” “gender-nonconformity” or “gender-nonconforming,” and “transperson.” The precision of each of these terms was calculated (Table 3). Records that included these search terms with a lower precision rate as compared to their respective databases were manually searched for other TGD-related terms that were also included in the search hedges in their titles, abstracts, and MeSH terms or keywords (Table 4). The aim was to see how much these individual search terms and phrases were contributing to less precise results, and how many of these irrelevant results would have been captured by each hedge if these terms were removed. The articles were further divided based on their include or exclude status at each screening phase.

**Table 3:** Breakdown of polysemic term prevalence in included and excluded records from MEDLINE and APA PsycInfo.

Term	Ovid MEDLINE			Ovid APA PsycInfo		
	INC	EXC	% INC	INC	EXC	% INC (INC/Total)
Gender inclusiv*	10	11	48%	13	15	46%
Gender divers*	136	33	80%	120	50	71%
**Gender diversity**	19	32	37%	36	46	44%
**Gender diverse**	120	3	98%	3	7	30%
Gender-nonconform*	37	11	77%	61	23	73%
Transperson*	1	17	6%	0	162	0%

INC: Include. EXC: Exclude.

**Table 4:** Co-occurrence of polysemic terms with other search terms in included records across MEDLINE and APA PsycInfo.

Term	Ovid MEDLINE			Ovid APA PsycInfo		
	TA	FT	Total	TA	FT	Total
Gender inclusiv*	6/7	2/3	8/10	11/11	1/2	12/13
**Gender diversity**	11/13	2/6	13/19	23/23	8/13	31/36
**Gender diverse**	NA	NA	NA	3/3	8/11	11/14
Transperson*	1/1	0/0	1/1	NA	NA	NA

TA: Title and abstract. FT: Full text.

## Results

**Fig 1:**
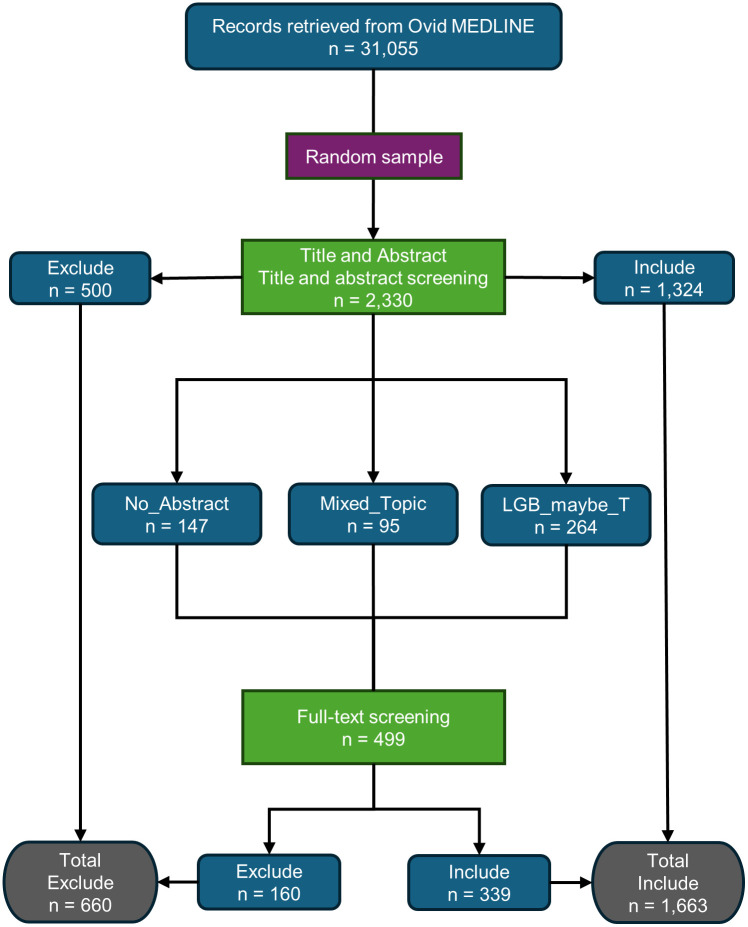
Validation process of the TGD search hedge for Ovid MEDLINE.

**Fig 2:**
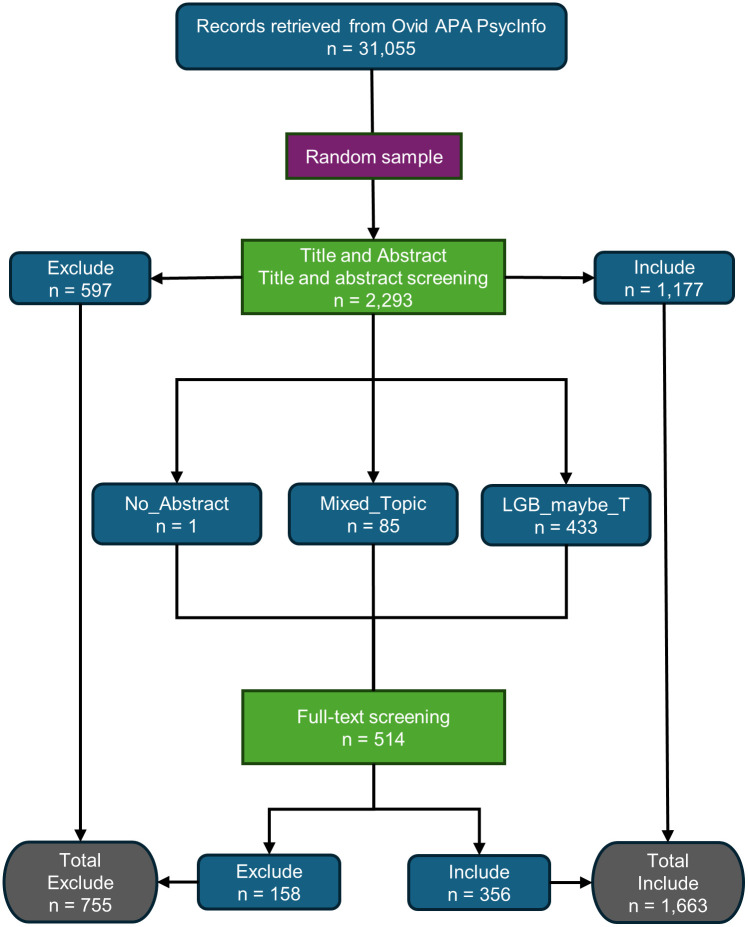
Validation process of the TGD search hedge for Ovid APA PsycInfo.

After improvements were made based on pilot testing, the final searches were conducted on June 7, 2024. A total of 30,055 articles were retrieved from Ovid MEDLINE (Fig 1), and 22,924 articles were retrieved from Ovid APA PsycInfo (Fig 2). A randomized sample of 2,330 articles were screened from Ovid MEDLINE and a random sample of 2,293 articles were screened from Ovid APA PsycInfo (for sample size justification see protocol [18]). In the title and abstract screening phase, 1,324 and 1,177 articles were categorized as relevant to TGD topics in Ovid MEDLINE and Ovid APA PsycInfo, respectively. In the full text screening phase, 339 and 356 articles were categorized as TGD-relevant in Ovid MEDLINE and Ovid APA PsycInfo, respectively.

Both the Ovid MEDLINE and Ovid APA PsycInfo search hedges identified all 144 of the 144 articles from the gold standard set of Knowsy 2STGNB evidence syntheses, resulting in 100% sensitivity in each database. The search hedges achieved a precision of 71% in Ovid MEDLINE and a precision of 67% in Ovid APA PsycInfo. These precision calculations were achieved through a total of 1,663 out of 2,330 articles deemed relevant to TGD populations in Ovid MEDLINE and 1,533 out of 2,293 articles deemed relevant to TGD populations in Ovid APA PsycInfo.

### Mixed_Topics and LGB_maybe_T categories

The majority of articles from Ovid MEDLINE classified as Mixed_Topics mentioned gender identity as part of social determinants of health, an equity-deserving group, or a possible focused topic for cultural competence training in education, health care, or the workplace. The term “gender non-conformity” was used in this category to describe externally observable behavior in children or adolescence without reporting on the participants’ gender self-identification. Similarly, another five articles discussed sex assignment surgery in Intersex people without consideration for the self-identification of the patients. Articles from APA PsycInfo in this category focused on theoretical discussion of gender identity, as well as the gender development of youth, intersex patients, and gay men. Less than half of the articles in this category were coded as TGD-relevant during full-text review (Table 2).

**Table 2:** Records included and excluded after full text review in each database stratified by ‘maybe’ classification type at title and abstract stage.

Category	Ovid MEDLINE			Ovid APA PsycInfo		
	INC	EXC	% INC (INC/ Total)	INC	EXC	% INC (INC/Total)
**Mixed Topics**	43	51	46%	35	50	41%
**LGB maybe T**	200	64	76%	320	108	75%

INC: Include. EXC: Exclude.

Overall, the majority of articles classified as LGB_maybe_T substantially discussed TGD people and/or topics. Articles from Ovid MEDLINE in this category discussed gender minority people, along with sexual minority people, in the context of equity consideration in education, healthcare, and training for service providers. While we have defaulted to TGD as an overarching category, gender minority and related phrases are part of the search hedges and are used widely in the extant literature. In addition to these topics, articles from APA PsycInfo discussed broader topics, including substance use and abuse, marriage equality, queer historical perspectives, and religion and spirituality. For both databases, the majority of the articles in this category were coded as TGD-relevant upon examination of the full-text (Table 2).

### Polysemic search terms

Specific search terms that had lower precision rates than their respective database precision included: “gender inclusiv*” in MEDLINE and APA PsycInfo (48% and 46%, respectively), “gender diversity” in MEDLINE and APA PsycInfo (37% and 44%, respectively), “gender diverse” in APA PsycInfo (30%), and “transperson*” in MEDLINE and APA PsycInfo (6% and 0%, respectively) (Table 3).

Most included articles also contained other search terms that allowed these articles to be picked up by the search hedge without the polysemic terms. “Gender inclusive,” “gender inclusivity,” and “gender diversity” were used in articles in MEDLINE pertaining to advocating for inclusion of TGD patients in medical and health settings. Similarly, in APA PsycInfo, these terms were used to discuss the experience of TGD people with discrimination in educational and professional settings. The only instance of “transperson*” that met inclusion criteria was used in the context of male-to-female transpersons. However, this article also contained the search term “transgender” in its title and abstract, as well as the MeSH heading term “transgender person” (Table 4).

The majority of excluded articles did not contain other search terms in the search hedges (Table 5). Excluded articles in both databases, use “gender inclusive” or “gender inclusivity” in a gender binary context, with frequent co-occurrence of the terms “male,” “female,” “women,” and “men.” These articles often focused on promoting greater presence of women in traditionally male-dominated fields (e.g., surgery, business) or advocating for better consideration of the experience of men in women-focused topics, such as domestic abuse and unintended pregnancy. For excluded articles in APA PsycInfo, “gender diversity*” and “gender diverse” have high co-occurrence rates. Similarly to their MEDLINE counterpart, articles with these terms often focused on promoting women and girls in sports, business, and education. In the sample used for validation, the term “transperson*” occurred exclusively in the context of transpersonal psychology in APA PsycInfo with no relevance to TGD topics, despite there being some relevant uses of the term “transperson*” in the full APA PsycInfo database. The hedge in the supplement has been modified to narrow matching terms to transperson or transpersons.

**Table 5:** Co-occurrence of polysemic terms and other search terms in excluded records across both databases.

Term	Ovid MEDLINE			Ovid APA PsycInfo		
	TA	FT	Total	TA	FT	Total
Gender inclusiv*	0/8	0/3	0/11	0/12	0/3	0/15
**Gender diversity**	1/22	0/10	1/32	6/6	6/19	12/25
**Gender diverse**	NA	NA	NA	6/6	4/5	10/11
Transperson*	0/16	0/1	0/17	0/162	0/0	0/162

TA: Title and abstract. FT: Full text.

Finally, the review process, which was conducted concurrently across both Ovid MEDLINE and Ovid APA PsycInfo, resulted in 642 disagreements in the title and abstract screening out of 4,623 records screened, meaning the rate of agreement across reviewers in aggregate was 13.9%. In the full text screening, 128 disagreements arose from the review of 1,013 full texts, resulting in a 12.6% rate of agreement. This indicates an improvement in agreement across reviewers in the later phase, despite the significant increase in material necessary to review in a full text when compared to a title and abstract record.

## Discussion

In this investigation, the performance of two search hedges were assessed for their ability to capture TGD-related literature in their respective databases, Ovid MEDLINE and Ovid APA PsycInfo. As comprehensive retrieval was the main priority, both searches included polysemic terms, creating a trade-off between decreased precision to increase sensitivity. The inclusion of these terms necessitated the creation of an additional category Mixed_Topics during the Title and Abstract Screening phase. Articles in the Mixed_Topics category often utilized the terms “gender inclusive,” “gender inclusivity,” or “gender diversity” in their titles and abstracts. Upon examination of the full text, these terms were used in the context of organizational efforts to include cisgender women and girls and not specifically TGD people. For example, one paper examined in this validation discussed and measured gender diversity in corporate governance boards using the Blau Diversity Index, which was defined as “$\text{1}\;-\;\sum_{i=\text{1}\;}^{n}\overset{\text{2}}{\underset{i}{p}}$, where $p_{i}$ is the percentage of board members in each category (two: male/female) and n is the number of categories” [34]. Thus, in this context, increasing gender diversity was understood as increasing the presence of cisgender women. In Ovid APA PsycInfo, the high co-occurrence of “gender diversity” and “gender diverse” in excluded articles suggested these two terms were used interchangeably to describe the concept of representation and equal opportunities for both cisgender men and women in different fields in the context of gender parity research.

In contrast, in Ovid MEDLINE, the term “gender diverse” yielded a high precision rate of 98%, suggesting that the term was used exclusively to refer to non-cisgender populations. The term “transperson” yielded low precision, only adding non-relevant articles to the result. The only relevant article containing the term “transperson” can also be retrieved by other terms in the hedges. Thus, “transperson” can be removed without decreasing the sensitivity of each hedge, depending on whether search hedge users prefer to prioritize precision or sensitivity.

The trade-off between sensitivity and precision is a common occurrence in the evidence synthesis process [35–37]. The current hedges can serve as a starting point for a comprehensive literature review on TGD populations for research projects such as evidence syntheses, rather than having to develop a search strategy from scratch or adapt existing unvalidated search strategies and expand upon them to fit the research question. Taken together, the precision of each noisy term offered adaptation guidance for future librarians to optimize the hedges based on the research question and the database. For example, if researchers want to prioritize precision and expediency, they could consider removing the term “gender inclusiv*,” “gender diversity,” and “gender diverse” in the Ovid APA PsycInfo hedge, as well as “gender inclusiv*” and “gender diversity” in the Ovid MEDLINE hedge.

Our development and validation process for the two hedges highlighted some common indexing patterns that raise methodological and epistemological concerns. In addition to creating the Mixed_Topics category, another category, LGB_maybe_T, was also created for the Title and Abstract Screening phase due to the difficulty in discerning whether an article containing the term “transgender” as a part of the LGBT acronym (and other variations) would be TGD-relevant. Further examination of articles in this illustrated a pattern of subsumption of the label “trans” into the broader array of the 2SLGBTIA+ acronym at the expense of specificity. This more expansive framing can help infer greater attention to sexual diversity and cisgender queer scholarship, as well as the research into these specific communities, while making it harder to locate research focused on trans and gender diverse people. Decisions on whether an article would offer substantive information regarding TGD people and/or topics could not be made based on the title and abstract and would have to rely on examination of the full text, thus making the knowledge/evidence synthesis process regarding TGD people and topics a more time and resource intensive process. This structural violence then confers additional burden on research into TGD people, adding to the on-going epistemic erasure of TGD people [38–40].

Further research quantitatively and conceptually deconstructing the use of the 2SLGBTQIA+ acronym and its relevance to TGD scholarly and scientific research would generate useful insights. Other teams or individual scholars interested in applying our hedges in their work should be aware of the potential for the larger 2SLGBTQIA+ acronyms to skew their research. In addition, authors submitting manuscripts should consider whether the work they publish is indeed relevant to the entirety of the 2SLGBTQIA+ population, or if it is perhaps more practical to use specific terms in keywords and indexing to avoid confusion. Further, while some hope that generative artificial intelligence will help researchers identify and synthesize the literature relevant to their research interests, the technology is not yet at the point where it can replace the performance of literature searching performed by human experts [41].This is especially true of literature pertaining to TGD populations, and likely for literature with other populations for whom terminology changes rapidly, or experience marginalization within academic research. Finally, the dynamic nature of language within communities, especially marginalized ones, warrants both special methodological consideration and caution. TGD populations may be motivated to shift language in an era of censorship and legislative attacks against them, and that language may or may not be reflected in the published, academic literature over time. In order to be inclusive, or rather to be especially sensitive in the case of our hedges, search strategies must be dynamic and include both current and historical terms. Search hedges, in our view, must be dynamic and therefore additive by nature not merely for the sake of inclusion, but also to avoid temporal bias which may result when problematic historical terms are removed from search hedges to appear more inclusive. For these reasons we advocate for both a culturally responsive approach, one that takes into account contemporary needs in order to respond to dynamics of language within TGD and other marginalized communities, and an additive one that does not dismiss potentially important information by removing terminology that may be out of date. At the same time, we suggest this approach to hedge construction, but insist that researchers adapt our hedge to their specific research questions, including removal of terms for tailoring if useful.

While these search hedges were designed with librarians and information scientists in mind, their utility extends to researchers across various disciplines. Not all research teams have access to highly trained librarians, and health scientists conducting systematic reviews, meta-analyses, or other forms of evidence synthesis may use the hedge to ensure comprehensive retrieval of literature related to transgender and gender diverse populations, particularly when investigating health disparities or access to gender-affirming care. Similarly, researchers in the social sciences and humanities may find the hedge valuable for identifying scholarship related to identity, structural marginalization, spatial belonging, and cultural specificity. Given the interdisciplinary nature of TGD research and the evolving language used to describe gender diversity, this hedge offers a practical tool for navigating inconsistent indexing and supporting transparent, reproducible search strategies.

### Limitations

Several important considerations should be noted when applying this hedge. First, as a product of its development by English language speakers and within North American academic contexts, the hedges reflect both linguistic and geographic bias. While it includes terms such as “Two-Spirit,” which hold cultural specificity, these terms do not necessarily align with Western constructions of transgender identity. They should not be assumed to do so. This underscores the importance of critical engagement with terminology, particularly when applying the hedge to research on culturally distinct gender identities. A strength of this study is that it is grounded in the community and that the majority of co-authors identify as TGD.

Second, these hedges were designed specifically for use in Ovid MEDLINE and Ovid APA PsycInfo. While they may be adapted for use in other databases, such as CINAHL, or other platforms, such as PubMed or EBSCOhost, care should be taken when translating syntax, field codes, and indexing conventions. It is also important to note that the syntax and field codes databases use are constantly evolving. For example, during the validation of these search hedges, the National Library of Medicine updated its thesaurus of medical subject headings (MeSH), updating the terminology used for two MeSH terms in our Ovid MEDLINE hedge. As of 2025, “Sex Reassignment Procedures” changed to “Gender-Affirming Procedures” and “Sex Reassignment Surgery” changed to “Gender-Affirming Surgery” in the National Library of Medicine thesaurus [30]. At the same time, new features may become available, such as APA PsycInfo adding a new population check tag during this study — “nonbinary.po” as a method of filtering search results to only include articles indexed as including non-binary participants [29]. While updated subject headings and new features to identify transgender or non-binary populations within databases are helpful, it is important to be aware of the limitations of relying on these strategies without a comprehensive search that is able to catch articles that are not indexed or indexed incorrectly.

Third, we validate these hedges against a gold standard data set which a co-author [REDACTED] created. As we have acknowledged elsewhere [18], this is a potential conflict of interest. In our view, it is not one because the librarian co-authors [REDACTED] and [REDACTED] discovered Knowsy independently when beginning to develop an early version of the hedges featured in this article. Collaboration with [REDACTED] was warranted given their expertise in the field and topic. That data set contained articles up to 2019; however, our searches were finalized in 2024. This could be interpreted as introducing a temporal bias because we cannot know how Knowsy would perform on the literature published between its end date in 2019 and our study’s end date in 2024. However, this is true of any gold standard data set.

Finally, users should note that terminology related to TGD identities continue to evolve. Language shifts — such as the introduction of “non-binary” or the declining use of terms like “transsexual” — require that the hedge remain dynamic. Accordingly, this is intended to be a living hedge. Librarian co-authors **Gagen** and **Marsalis** will maintain and periodically update the hedge to reflect language, indexing practices, and community usage developments as part of the dissemination plan via platforms frequently consulted by expert researchers in the health and social sciences.

## Supporting information

S1 File Search Hedges for Ovid MEDLINE and Ovid APA PsycInfo. (DOCX)
