# Supplementary material for: Development and validation of search hedges for Transgender and Gender Diverse (TGD) populations in Ovid MEDLINE and Ovid APA PsycInfo
Source: PLoS One. 2026 Jun 12;21(6):e0351303. doi: 10.1371/journal.pone.0351303 (PMC13262833; doi:10.1371/journal.pone.0351303)
Supplement: S1 File — (DOCX) [file pone.0351303.s001.docx]

## Supporting Information – S1 Search Hedges

Search hedges as updated as of February 2026. See notes for changes from validated versions. All phrases have been enclosed in straight quotation marks to facilitate translation to other platforms.

### Ovid MEDLINE search hedge

1 exp Gender-Affirming Care/ or exp Gender-Affirming Procedures/ or exp Gender-Affirming Surgery/ or exp Gender Dysphoria/ or exp Gender-Nonconforming Persons/ or exp Health Services for Transgender Persons/ or exp "Sexual and Gender Disorders"/ or exp Transgender Persons/ or exp Transsexualism/ or exp Transvestism/

2 ("trans bod*" or "trans elder*" or "trans experienc*" or "trans folk*" or "trans folx*" or "trans gender*" or "trans ident*" or "trans individual*" or "trans parent*" or "trans people*" or "trans person*" or "trans selv*" or "trans senior*" or "trans sex*" or "trans spectrum" or "trans visib*" or "trans youth*" or transex* or transfolk* or transfolx* or transgender* or transident* or transpeople* or transperson* or transsex* or transvesti* or transvisib*).ti,ab,kf,kw,ot,oa,cl.

3 (AFAB or "assigned female" or "trans boy*" or "trans father*" or "trans guy*" or "trans male*" or "trans man" or "trans masc*" or "trans men" or transboy* or transguy* or transmale* or transman or transmasc* or transmen).ti,ab,kf,kw,ot,oa,cl.

4 (AMAB or "assigned male" or "trans female" or "trans femin*" or "trans femme*" or "trans girl*" or "trans mother*" or "trans woman" or "trans women" or transfemal* or transfemin* or transfemme* or transgirl* or transwoman or transwomen).ti,ab,kf,kw,ot,oa,cl.

5 ("2 spirit*" or agender* or ((androgynous or androgyny) not bem*) or "atypical gender" or "bi gender*" or bigender* or "demi boy*" or "demi girl*" or demiboy* or demigirl* or "dissident gender" or "gender atypical*" or "gender bend*" or "gender binar*" or "gender creativ*" or "gender divers*" or "gender expans*" or "gender expression*" or "gender fluid*" or "gender flux*" or "gender identit*" or "gender inclusiv*" or "gender minorit*" or "gender modalit*" or "gender non conform*" or "gender nonconform*" or "gender queer*" or "gender questioning" or "gender varian*" or genderdivers* or genderexpans* or genderflu* or genderqueer* or genderquestioning or "minority gender*" or neutrois or "non binary*" or "non cisgender*" or nonbinar* or noncisgender* or (TGD not yangtze) or thirdgender* or "third gender*" or "third sex" or "third spirit*" or trigender or "two spirit*" or twospirit*).ti,ab,kf,kw,ot,oa,cl.

6 ((acault and myanmar) or achout or aikane or "akava ine" or "akava’ine" or alyha or aravani or aravanis or ashtime or bakla or bantut or basivi or berdache* or bissu or "brother boys" or brotherboys or burrnesha or calabai or calalai or dilbaa or "fa afafine" or "fa’afafine" or fakafefine or fakafifine or fakaleiti or femminiell* or guevedoche or hijra* or hirja* or hwame or irahuhua or irawhiti or kathoe* or kathoey or kathoy or katoey or khanith or "khwaja saraa" or "khwaja sira" or kocek or kothi or koti or ladyboy* or leiti or lhamana or machi or mahu or mahuvahine or mahuwahine or "mak nyah" or maknyah or mashoga or ((meti or metis) and Nepal) or muxe or muxes or muxhe or nadleehi or ninauposkitzipxpe or paknyah or palopa or panthi or "phuying kham phet" or pinapinaaine or quariwarmi or "sao praphet song" or sekrata or selrata or "sister girls" or sistergirls or skoptsy or "sworn virgin" or "sworn virgins" or tahine or takataapui or "tangata ira tane" or "tangata ira wahine" or transpinay* or transpinoy or travesti* or vakasalewalewa or wakatane or waria or whakawahine or winkte or xanith).ti,ab,kf,kw,ot,oa,cl.

7 (autogynephil* or "cross gender*" or "cross sex hormon*" or crossgender* or "gender adjustment" or "gender affirm*" or "gender chang*" or "gender confirm*" or "gender disorder*" or "gender dysphor*" or "gender euphor*" or "gender identity disorder*" or "gender incongruen*" or "gender re-assign*" or "gender reassign*" or "gender transition*" or "sex affirm*" or "sex chang*" or "sex re-assign*" or "sex reassign*" or "sex transition*" or "sexual dysphor*" or "sexual reassignment" or "trans affirm*" or transaffirm* or "facial feminis*" or "facial feminiz*" or "facial masculinis*" or "facial masculiniz*" or "genital reconstruct*" or genitoplasty or metoidioplasty or neophallus or neovagina or phalloplasty or "pregnant man" or "pregnant men" or "puberty block*" or "puberty suppress*" or vaginoplasty or "vocal feminis*" or "vocal feminiz*" or "vocal masculinis*" or "vocal masculiniz*").ti,ab,kf,kw,ot,oa,cl.

8 ("cross dress*" or crossdress* or "drag king*" or "drag queen*" or "female impersonat*" or "male impersonat*").ti,ab,kf,kw,ot,oa,cl.

9 (misgender* or tranny or transphobi*).ti,ab,kf,kw,ot,oa,cl.

10 (gender neutral adj5 (pronoun* or language or bathroom*)).ti,ab,kf,kw,ot,oa,cl.

11 1 or 2 or 3 or 4 or 5 or 6 or 7 or 8 or 9 or 10

12 11 not ((exp chordata/ or exp chordata, nonvertebrate/ or exp amphibians/ or exp birds/ or exp fishes/ or exp afrotheria/ or exp artiodactyla/ or exp carnivora/ or exp cetacea/ or exp chiroptera/ or exp eulipotyphla/ or exp lagomorpha/ or exp pangolins/ or exp perissodactyla/ or exp rodentia/ or exp scandentia/ or exp xenarthra/ or exp marsupialia/ or monotremata/ or reptiles/ or exp invertebrates/ or exp choanoflagellata/ or exp cryptophyta/ or exp diplomonadida/ or exp euglenozoa/ or exp fungi/ or exp glaucophyta/ or exp haptophyta/ or exp mesomycetozoea/ or exp oxymonadida/ or exp parabasalidea/ or exp plants/ or exp retortamonadidae/ or exp rhizaria/ or exp rhodophyta/ or exp stramenopiles/ or exp viridiplantae/ or exp archaea/ or eukaryota/ or exp alveolata/ or exp amoebozoa/ or exp choanoflagellata/ or exp cryptophyta/ or exp diplomonadida/ or exp euglenozoa/ or exp fungi/ or exp glaucophyta/ or exp haptophyta/ or exp mesomycetozoea/ or exp oxymonadida/ or exp parabasalidea/ or exp plants/ or exp retortamonadidae/ or exp rhizaria/ or exp rhodophyta/ or exp stramenopiles/ or exp viridiplantae/ or exp archaea/ or exp bacteria/ or exp viruses/ or exp organism forms/) not humans/)

**Notes on Ovid MEDLINE search hedge:**

- Line 1 (MeSH terms): "Sex Reassignment Procedures" changed to "Gender-Affirming Procedures" and "Sex Reassignment Surgery" changed to "Gender-Affirming Surgery" with 2025 MeSH update. "Gender-Affirming Care" and "Gender-Nonconforming Persons" are MeSH terms added in 2024 that have been added to the search hedge.

### Ovid APA PsycInfo search hedge

1 Drag performance/ or Gender Affirming Care/ or Gender dysphoria/ or Gender expression/ or Gender Nonbinary/ or Gender nonconforming/ or Gender reassignment/ or Transgender/ or exp "Transgender (Attitudes Toward)"/ or Transsexualism/ or Transvestism/ or Two-Spirit/

2 (gender identity/ or lgbtq/ or "gender identit*".tw.) and (transgender.po. or nonbinary.po)

3 ("trans bod*" or "trans elder*" or "trans experienc*" or "trans folk*" or "trans gender*" or "trans identi*" or "trans individual*" or "trans parent*" or "trans people*" or "trans person*" or "trans selv*" or "trans senior*" or "trans sex*" or "trans spectrum" or "trans visib*" or "trans youth*" or transex* or transfolk* or transgender* or transident* or transpeople* or transperson? or transsex* or transvesti* or transvisib*).tw.

4 (AFAB or "assigned female" or "trans boy*" or "trans father*" or "trans male*" or "trans man" or "trans masc*" or "trans men" or transboy* or transmale* or transman or transmasc* or transmen).tw.

5 (AMAB or "assigned male" or "trans female" or "trans femin*" or "trans femme" or "trans girl*" or "trans mother*" or "trans woman" or "trans women" or transfemal* or transfemin* or transfemme* or transgirl* or transwoman or transwomen).tw.

6 ("2 spirit*" or agender or ((androgynous or androgyny) not bem*) or "atypical gender" or "bi gender*" or bigender* or "demi boy" or "demi girl" or demiboy or demigirl or "dissident gender" or "gender atypical*" or "gender bend*" or "gender binar*" or "gender creativ*" or "gender divers*" or "gender expans*" or "gender expression*" or "gender fluid*" or "gender flux*" or "gender inclusiv*" or "gender minorit*" or "gender modalit*" or "gender non conform*" or "gender nonconform*" or "gender queer*" or "gender questioning" or "gender varian*" or genderdivers* or genderexpans* or genderflu* or genderqueer* or genderquestioning or "minority gender*" or neutrois or "non binar*" or "non cisgender" or nonbinar* or noncisgender or (TGD not yangtze) or "third gender*" or "third sex" or "third spirit*" or trigender or "two spirit*" or twospirit*).tw.

7 ((acault and myanmar) or achout or aikane or "akava ine" or "akava'ine" or alyha or aravani or aravanis or ashtime or bakla or bantut or basivi or berdache* or bissu or "brother boys" or brotherboys or burrnesha or calabai or calalai or dilbaa or "fa afafine" or "fa'afafine" or fakafefine or fakafifine or fakaleiti or femminiell* or guevedoche or hijra* or hirja* or hwame or irahuhua or irawhiti or kathoe* or kathoey or kathoy or katoey or khanith or "khwaja saraa" or "khwaja sira" or kocek or kothi or koti or ladyboy* or leiti or lhamana or machi or mahu or mahuvahine or mahuwahine or "mak nyah" or maknyah or mashoga or ((meti or metis) and Nepal) or muxe or muxes or muxhe or nadleehi or ninauposkitzipxpe or paknyah or palopa or panthi or "phuying kham phet" or pinapinaaine or quariwarmi or "sao praphet song" or sekrata or selrata or "sister girls" or sistergirls or skoptsy or "sworn virgin" or "sworn virgins" or tahine or takataapui or "tangata ira tane" or "tangata ira wahine" or transpinay* or transpinoy or travesti* or vakasalewalewa or wakatane or waria or whakawahine or winkte or xanith).tw.

8 (autogynephil* or "cross gender*" or "cross sex hormon*" or crossgender* or "gender adjustment" or "gender affirm*" or "gender chang*" or "gender confirm*" or "gender disorder*" or "gender dysphor*" or "gender euphor*" or "gender identity disorder*" or "gender incongruen*" or "gender re-assign*" or "gender reassign*" or "gender transition*" or "sex affirm*" or "sex chang*" or "sex change operation*" or "sex re-assign*" or "sex reassign*" or "sexual dysphor*" or "sexual reassignment" or "trans affirm*" or transaffirm*).tw.

9 ("facial feminis*" or "facial feminiz*" or "facial masculinis*" or "facial masculiniz*" or "genital reconstruct*" or genitoplasty or metoidioplasty or neophallus or neovagina or phalloplasty or "pregnant man" or "pregnant men" or "puberty block* puberty suppress*" or vaginoplasty or "vocal feminis*" or "vocal feminiz*" or "vocal masculinis*" or "vocal masculiniz*").tw.

10 ("cross dress*" or crossdress* or "drag king*" or "drag queen*" or "female impersonat*" or "male impersonat*").tw.

11 (misgender* or tranny or transphobi*).tw.

12 (gender neutral adj5 (pronoun* or language or bathroom*)).tw.

13 or/1-12

**Notes on Ovid APA PsycInfo search hedge:**

- Line 2: "Nonbinary.po" added as APA PsycInfo population group in 2025. "Transgender.po" and "nonbinary.po" population groups are combined with keywords and not searched as standalone terms due to being overly sensitive.
- Line 3: "Transperson*" changed to "transperson?" (0 or 1 character wildcard) to exclude irrelevant results stemming from the term "transpersonal" in APA PsycInfo.
