# Supplementary material for: Development and validation of search hedges for Transgender and Gender Diverse (TGD) populations in Ovid MEDLINE and Ovid APA PsycInfo
Source: PLoS One. 2026 Jun 12;21(6):e0351303. doi: 10.1371/journal.pone.0351303 (PMC13262833; doi:10.1371/journal.pone.0351303)
Supplement: Attachment — Submitted filename: response_to_reviewers.docx [file pone.0351303.s003.docx]

**Manuscript PONE-D-26-08417**

**Response to Reviewers**

Dear Dr. Roberts & PLoS One,

Thank you for the opportunity to submit a revised draft of our manuscript “Development and validation of search hedges for transgender and gender diverse populations in Ovid MEDLINE and Ovid APA PsycInfo” for publication in *PLoS One.* We appreciate the time and effort you, your editorial staff, and the reviewers have committed to offering constructive feedback on our article. We have incorporated many of the suggestions made by reviewers, especially those that appear in multiple reviewers’ feedback and are underscored by yourself as of particular import. Those changes are highlighted in the tracked changes version of the manuscript we have submitted. Below, we offer responses, in black, to each piece of feedback from reviewers, in red, along with any editorial comments, in blue, and organized by the order in which they appeared in the combined feedback we received. We look forward to consideration of our revised manuscript and are grateful for the opportunity.

**Reviewers’ Comments to the Authors:**

***Reviewer 1:***

The manuscript would benefit from more explicit discussion of how the search hedges translate across databases. Because indexing systems, controlled vocabularies, and field structures vary substantially between databases, readers would benefit from guidance on:
* which elements of the hedge are database-specific,

* how subject headings were mapped across databases, and

* what adaptations are required for implementation in other search platforms.

*Editor's elaboration:*

You touch on this issue in your manuscript, but provide minimal details:

**Line 115:** *"Although these hedges are designed and validated on the Ovid platform, they are constructed in such a way to facilitate translation to other platforms, including PubMed, EBSCOhost, ProQuest, and APA PsycNet."*

You can expand on this statement in your manuscript if you want. However, I am concerned that a review of how to adapt search hedges for use in different databases is beyond the scope of this manuscript. You can consider providing a reference to an article discussing this topic as an alternative to the detailed discussion suggested by Reviewer 1.

Author response:

The following text has been added to the first paragraph of subsection “Search hedge structure” under the Methods section and appears beginning at line 179 in the manuscript.

Search hedges can be translated across different databases and between different interfaces and platforms that search the same database. Glanville et al. (2019) provide a case study detailing how to translate a validated search hedge from one platform to another.

For ease of translation across different databases, the controlled vocabulary terms for each search hedge have been grouped in the first line of each respective hedge. These database-specific controlled vocabulary terms are the MeSH terms for Ovid MEDLINE and the APA Thesaurus of Psychological Index terms for Ovid APA PsycInfo. The other database-specific elements that would need to be translated for other databases or platforms would be the field codes (such as ".ti,ab,kf,kw,ot,oa,cl." or ".tw.") and the adjacency operator ("adj5") that we tried to minimize.The human limiter for Ovid MEDLINE (line 12) is specific to that database to mitigate indexing errors. We tried to limit the need to translate other database-specific syntax such as advanced wildcard operators to reduce potential errors in translation. In addition, we address this in the limitations section in the original manuscript and have kept it in this revision.

**Line 63:** *"When utilizing a search hedge, information professionals and researchers should consider if the hedge has been validated, if it optimizes sensitivity or precision, when it was last updated, and whether it needs to be adapted to suit their specific research question."*

This is a useful recommendation, but it would benefit from contextualization. The authors might discuss how often validated search hedges exist in practice, in which fields they are most common (e.g., clinical medicine vs. social sciences), and for approximately how many types of search queries such hedges have been developed.

**Editor's elaboration:**

I feel Reviewer 1's suggestion has merit and your paper would benefit from additional contextualization. However, I am concerned that a review of search hedges use is beyond the scope of this paper. Consider directing readers to papers addressing this general information about search hedge use, such as reference 4, as an alternative to a full discussion.

Author Response:

We agree that some more context about utilizing search hedges would be beneficial to our paper, although we share the editor's point that some of the points Reviewer 1 brought up are outside the scope of this paper.

To address this concern we revised the introduction section in several places. First, our second paragraph now contains further information about the utility of search hedges, generally (lines 62-63 and 65-69). Second, we expanded upon the paragraph this reviewer cites beginning just after the sentence they quote (lines 87-). The text for each of these revisions appears below:

62-63 “...as pre-constructed components of a larger, comprehensive search strategy in evidence synthesis reports…”

Lines 65-69 “In particular, search hedges can improve the comprehensiveness and sensitivity of database queries when searching for complex concepts and populations that have significant language variability. Researchers can incorporate search hedges as an integral part of their approach to evidence synthesis, thus making their searches more complete and reducing the risk of missing relevant literature.”

Lines 87-92 “While a more thorough explanation of search hedges as tools for more general use is beyond the scope of this study, others have described the landscape of existing topic search hedges. There are a variety of sources for validated search hedges. For example, the InterTASC Information Specialists’ Sub-Group Search Filter Resource provides access to published and unpublished search hedges as well as information about how to critically develop and critically appraise search hedges.

**Line 442:** *"Librarian co-authors [REDACTED] and [REDACTED] will maintain and periodically update the hedge to reflect language, indexing practices, and community usage developments."*

It would be helpful to specify where these updates will be maintained. The authors might consider discussing whether a platform could be used to host and version search hedges. For example, research infrastructures or methodological repositories could serve as dissemination platforms. In the digital humanities and social sciences, infrastructures such as the *SSH Marketplace* operated by *DARIAH-EU* publish methodological resources; a similar approach could potentially be used for search hedges

**Line 89:** "Through active maintenance and user adaptation, these hedges should be refined and expanded to reflect changes in language, indexing preferences, and the needs of researchers working with TGD populations over time."

The authors should clarify whose responsibility such maintenance would be, how updates might be coordinated, and how frequently revisions should occur.

**Editor's Elaboration:**

I agree that it is important to update search hedges to *"to reflect language, indexing practices, and community usage developments"* and to ensure dissemination of these search hedges for use by others. However, I feel your paper should focus on the work being presented rather than describing work you may do in the future. Consider limiting your discussion to the need for this work to occur in the future. You could also direct readers to any currently available dissemination platforms for search hedges via a reference, in the manuscript itself, or via a supplemental appendix.

Author response:

Thank you for the suggestion to add information about search hedge dissemination platforms. See our comment above for the language we added that directs readers to an example of a repository for existing search filters. As discussed by the editor, we wanted to focus on the presentation of our current work rather than describing potential future work. We also added to the final sentence of the manuscript, which this reviewer cites, to name that ongoing updates are part of our dissemination plan.

The manuscript would benefit from greater clarity and consistency in terminology. The text alternates between several related but distinct terms such as “transgender and gender diverse”, “transgender”, “diverse and expansive gender identities”, and “2SLGBTQIA+”. These terms refer to overlapping but not identical populations and conceptual frameworks. The authors should define these terms clearly and justify their choice of terminology in relation to the scope of the search hedges.

Additionally, it should be acknowledged that some less advantaged communities do not prefer the term “minority”.

Author response:

Throughout the article, we refer to the population the hedges are about as "transgender and gender diverse" wherever possible to acknowledge that the scope of our search hedges are designed to capture transgender people in addition to gender diverse terminology for people who may or may not consider themselves transgender, but who do not identify as their assigned sex at birth.

When we refer to "2SLGBTQIA+," we are discussing the larger spectrum of sexuality and gender identity which provides unique challenges for identifying literature on transgender and gender diverse populations.

We acknowledge that many of the terms used throughout the search hedges and in this article may be antiquated or offensive. Searches supporting evidence syntheses require robust and comprehensive searches to identify all potentially relevant literature. We have also revised the few places where we used the minority language ourself to either clarify our intention, i.e., discussing specific results, or to change it to our TGD overarching terminology.

The manuscript refers to validation using sensitivity and precision, but it would be clearer to use the more established information-retrieval terminology of *recall* and *precision*. The authors note that: "search hedges are validated by testing their sensitivity, also referred to as recall, against a 'gold standard' set of known relevant records."

A reference should be provided for this methodological approach, or the authors should verify whether a canonical reference (e.g., from information science or systematic review methodology literature) exists. The statement that hedges are evaluated by: "their precision, which is the ratio of relevant to irrelevant records retrieved [6]. A challenge of search hedge development is balancing sensitivity and precision, as they are inversely related" is correct but would benefit from clearer explanation of how this trade-off was operationalized in this study.

Author response:

We initially name sensitivity on line 76 in the original manuscript and in the same sentence state that it is also referred to as recall. We’ve now added citations [6,7] to this sentence to indicate our decision to use sensitivity is consistent with Campbell and Cochrane guidance.

"The hedges were validated for sensitivity using a gold standard set of 144 articles from the Knowsy portal of evidence syntheses tagged as Two-Spirit, transgender, or gender non-binary."

However, further methodological detail is required. For example:

- How were the 144 articles selected and curated in the Knowsy portal?

- Why was this particular dataset chosen as the gold standard rather than other potential sources?

…

The authors should also acknowledge limitations of relying on validation against a gold standard dataset. For example:

* new articles published after the creation of the gold standard may not be represented,

* the gold standard itself may omit relevant studies,

* validation results may therefore underestimate or overestimate actual recall.

Author response:

We appreciate the reviewer’s feedback and concerns. And while the methodological choice to rely on Knowsy as a gold standard set reflects upon this article, the methods behind that data set are not part of this study. In addition, there are no alternatives that our author team are aware of that are comprehensive and not delimited by other hedge portions (such as a full PICO). Below we address these concerns as they pertain to the study at hand.

There are multiple approaches to identifying a gold standard dataset, predominantly hand searching and relative recall, which we addressed in lines 78-80 of the original manuscript and have left in the revision. We have now added a citation to that sentence that addresses the use of gold standard sets for validation. Before addressing the specifics of our hedge and the gold standard set we used to validate it, we want to also address this reviewer’s bulleted comments about limitations. These are true of all gold standard sets (temporal limits, omission, under/overestimation). While our measure of sensitivity is based largely on Knowsy, we validated a large random sample of all records returned from our hedge in both databases. The existing explanation in our methods of that process, in our view, demonstrates that our methods for validation did not rely solely on a gold standard set, but rather on a robust process for screening and review.

The Knowsy dataset was created from searches of MEDLINE, APA PsycInfo, Embase, and CINAHL.designed by a librarian not involved in this study. The Knowsy team manually screened records and assigned population tags. Thus, we were able to utilize an existing dataset created independently from the librarians who developed these search hedges. For the current gold standard dataset, we used all studies tagged with the terms gender identity, Two-Spirit, transgender, or gender non-binary.

Moreover, for this specific hedge, which is focused on TGD populations, there is good reason to use an ‘older’ dataset as a gold standard. Because of the lag between the introduction of new terminology that TGD communities actually use and publication that deploys that terminology in the lieterature, we would be more concerned with sensitivity for a dataset that is older. This is because we would want to make sure that our hedge is as comprehensive and temporally unbiased as possible. This also led us to leave in many of the terms that didn’t necessarily have any unique hits so that it can be an evolving search hedge and capture new things as they are being published. On the other end of the spectrum, sensitivity to a gold standard, in our case Knowsy, that included all literature up to 2014 would indicate that our hedge performs well with historical data as well, which makes it more useful to a wider array of researchers and scholars, including those working in history and related fields who may be more concerned with a larger temporal period than just the most up to date evidence available.

More detail is also needed regarding the screening process described in the manuscript:

"an international research team of subject experts independently screened a randomized sample of search results in a two-stage screening process with an additional screener resolving disputes."

The manuscript should specify how many reviewers were involved, how disagreements were resolved, and whether inter-rater agreement or another measure of consistency was assessed.
…"the majority of the articles in this category were coded as TGD-relevant upon examination of the full-text" The criteria used to determine relevance should be clearly defined, and the procedures for resolving disagreements should be described.

Author response:

Members of the author team for this study have already published a protocol paper on our methods, which we cite. While we recognize that this article under consideration should stand on its own, we do consider some of the details to be out of scope. In that protocol paper we outline the screening process thoroughly and share only the most immediately relevant details here, which is not an uncommon practice in scientific literature. We have, however, included a interrater reliability measure at the reviewer’s suggestion in hopes that it clarifies the effectiveness of our approach. And, where relevant, we have added further language to explain the inclusion criteria, while still directing readers to the protocol paper should they be especially interested in the details of our screening process.

More specifically, we have added further elaboration in the final paragraph of the “Validation” section of the Methods. In addition, we added some further language to clarify what constituted grounds for inclusion as TGD relevant. Finally, we have reported on agreement rates during both phases of screening to indicate how frequently reconciliation by consensus among study team members was necessary to resolve conflicts in screening records. There was a slight improvement in the full text phase from the title and abstract phase.

The authors should consider whether Homosaurus, a controlled vocabulary widely used for LGBTQ+ information organization, could have been used to identify relevant search terms. If it was considered but not used, the manuscript could explain why. Given that the study addresses evolving terminology, engagement with existing domain-specific vocabularies would strengthen the methodological justification.

Author response:

Thank you for your feedback. Our team is aware of Homosaurus as an important international resource for LGBTQ+ vocabulary. Homosaurus is a tool primarily used alongside Library of Congress Subject Headings for use in cataloging and archival work to improve LGBTQ+ subject vocabulary in MARC records. Search hedges are developed for comprehensive database searches primarily for evidence synthesis methodologies. Despite being an incredibly valuable data source, Homosaurus is not particularly well-suited for ease of use in comprehensive literature searches.

**Some sentences require clarification or expansion.** For example, the manuscript states:

**Line 59:** *"Social science journals have been slower to adopt structured abstracts which can be a barrier to efficient search development [7,8]."*

In its current form, this sentence appears disconnected from the surrounding paragraph. The authors should either expand on how the absence of structured abstracts affects search hedge development or remove the sentence.

Author response:

Thank you; we agree that this sentence doesn't really add to the argument and has been deleted. The original references 7 and 8 are also removed accordingly

Similarly, the statement: **Line 60:** *"Reviews on topics with developing or dynamic terminology, especially require a more sensitive strategy."* is unclear and should be elaborated. The authors could specify why dynamic terminology—such as evolving gender identity terms—creates challenges for search strategy construction and why higher recall may be necessary in such contexts.

Author response:

We appreciate the reviewer’s suggestion to clarify this. We have opted to incorporate this into the discussion section, rather than in the introduction.

See lines 483-497 for further details, but, in brief, we suggest search hedges like this should be both dynamic and additive and individual researchers can tailor their use of our hedge to their specific needs.

**Line 69:** *"Existing search strategies often fail to capture the full range of gender diversity — particularly when it comes to culturally specific gender identities, Indigenous systems of sex and gender, and evolving gender terminology."*

This is an important point but requires additional references and engagement with existing research on the discoverability of LGBTQ+ scholarship and culturally specific gender identities in bibliographic databases.

Author response:

We appreciate the reviewer’s feedback. We do not speak to the broader discoverability of LGBTQ+ research and scholarship specifically because we find that it is not always actually inclusive of the ‘T.’ This is our motivation for the unorthodox approach to screening wherein we had a maybe category that was specifically about the use of the LGBT acronym in a way that warranted investigation at full text to determine if TGD populations were actually discussed or merely gestured toward with an acronym.

In addition, we do not address the discoverability of culturally specific gender identities in bibliographic databases because, to the best of our knowledge, peer-reviewed scholarship in this area does not exist. There is, of course, a large and ever-growing body of literature on culturally specific gender identities. But to our knowledge, no study thus far has collated them and discussed their discoverability in bibliographic databases. Instead, we have added language and a citation specifying the challenges that two-spirit in particular poses to traditional knowledge organization systems, and a note about a dearth of research on discoverability to the section this reviewer quotes.

**Line 78:** *"while other terms that do not currently retrieve many records have been deliberately included for future growth as scholarly research adapts to emerging terminology"*

The manuscript should clarify what “future growth” refers to and whose needs this anticipates (e.g., future researchers, evolving academic terminology, indexing updates). Providing concrete examples of such terms would improve clarity.

Author response:

Some of the current terminology utilized in the search hedges, such as demiboy, genderflux, "trans folx," "trans guy," and others currently receive no results in either Ovid MEDLINE or Ovid APA PsycInfo.

As experts on transgender and gender diverse literature, we left in terminology that we thought may appear in relevant literature in the near future to anticipate the needs of future researchers.

***Reviewer 2:***

**Lines 77-78:** I found the sentence structure difficult here. Consider changing to "Reviews on topics with developing or dynamic terminology require a more sensitive strategy."

Author response:

Thank you, this sentence has been clarified.

**Lines 79-80:** Suggest adding consideration of which database platform the hedge is for

Author response:

Added clarification that we deliberately included terms that currently do not retrieve many records *in Ovid MEDLINE or Ovid APA PsycInfo*. This should provide clarity about the platform and databases these hedges are developed for.

**Line 93:** remove the "s" from the end of 'retrieves' as it is referring to 'search hedges'

Author response:

Thank you, this grammatical error has been corrected.

**Line 145:** Consider defining the term "cisgender" when it first appears

Author response:

Great suggestion! A definition of cisgender has been added to the first time it appears in the manuscript. The sentence now ends "... cisgender people — non-transgender individuals whose current gender identities correspond to the sexes they were assigned at birth."

**Line 449:** Remove "At the same time" from beginning of sentence as it is the beginning of a new section and reads as a continuation of a paragraph instead.

Author response:

Thank you, the sentence that begins the "Limitations" section has been edited to remove "At the same time." The sentence now reads, "Several important considerations should be noted when applying this hedge."

***Reviewer 3:***

"In your previous published protocol, your team acknowledged a possible conflict of interest with two authors of the protocol overlapping with Knowsy. This might need to be acknowledged in this manuscript as well."
**Editor's elaboration:** Please include this in your discussion

Author response:

We have edited the discussion to include a potential conflict of interest in the limitations section.

**Line 118:** Methods section should have a subheading for the Gold Standard Set. The gold standard set--what it includes, how it was developed, why it was selected, and why it is appropriate--are all very important to the validation methods and resulting outcomes. This should be described in detail. Would also be good to briefly discuss external validation vs internal as you did in the protocol.

Author response:

We appreciate the suggestion from this reviewer to add a subheading about the gold standard set. However, the purpose of publishing our protocol paper was not only for transparency about our methods, but also to be able to direct readers to it who may be interested in replicating the study design or simply in understanding the methods. We do not wish to risk significant overlap with an existing published article, or even worse, risk potential self-plagiarism for members of the author team, and thus have chosen to address some of the reviewers’ questions about Knowsy in-text within the existing structure.

**Line 135:** In the “search term selection” section, you state that “we consulted the initial searches used to populate Knowsy,” but I don’t see that this list is publicly available on the Knowsy website, but I may have missed it. Please specify if you got the list from the public website or if you got the list from one of the co-authors in this manuscript who helped to develop or continue to maintain Knowsy. In your previous published protocol, your team acknowledged a possible conflict of interest with two authors of the protocol overlapping with Knowsy. This might need to be acknowledged in this manuscript as well.

Author response:

We have acknowledged this overlap and potential conflict of interest in the limitations section of our discussion. The studies in the Knowsy gold standard data set are from the 2019 iteration and are publicly available via the website.

**Additional Limitations:** Your previously published protocol provides more information about the Knowsy gold standard set of articles, which were collected in 2019. This should be acknowledged as a limitation because it means that your gold standard set does not include recent articles published between 2019-2026, and that your search hedge reflects this. This can be connected back to your discussion regarding the need to consistently update due to evolving language and database functionality and goal of continuing to update and maintain this hedge.

Author response:

We appreciate this suggestion, thank you. The additional paragraph in our limitations section now addresses the temporal mismatch of our searches and the Knowsy gold standard.

**Line 291:** "It looks like Table 3 is listed in the manuscript before Table 2. Not sure if this matters for the journal formatting, but pointing it out just in case. It does refer to the correct table so is probably okay.
**Editor's elaboration:** Please number your tables in the order they appear in the manuscript.

Author response:

Thank you, we have revised the table numbers in the order they appear in the manuscript.

**Lines 74-76:** The terms precision and sensitivity can be further defined to provide clarity. I recommend incorporating a brief summary of your definitions of these terms in your previously published protocol.

Author response:

Thank you for this suggestion. It was also made by reviewer 1. We have clarified above in response to reviewer 1 where we have made revisions to address this concern about precision and sensitivity. We have added citations as well that emphasize the importance of sensitivity and why we use this term over ‘recall.’

**Lines 96-98:** The term “excessive noise” should be defined in terms of what it actually means. In this context, you are using “excessive noise” as a metaphor and not everyone reading this will understand what you mean. You use similar terms (e.g., noisy) throughout the manuscript. Either define it the first time you use it so that it is clear what you mean, or use more precise non-metaphorical descriptions.

Author response:

Thank you for the suggestion. We added a brief parenthetical description after our first use of "noise" in our manuscript, lines ~77-78: "Terms that produced excessive noise (irrelevant search results)..."

**Line 115:** This is the first time you mention Medline and PsycInfo in the main text (other than the title and abstract). Include Ovid here like you do in the title and abstract just to make it clear that this is the platform you used.

**Line 377:** Again, specify OVID when naming the databases.

Author response:

Thank you, we have replaced all instances of "MEDLINE" with "Ovid MEDLINE" and all instances of "APA PsycInfo" with "Ovid APA PsycInfo" for clarity that both databases were searched via the Ovid platform.

**Lines 140-143:** This last sentence (starting with “cultural specific terms” is a little confusing and could be revised into more simple or straightforward terms for clarity.

Author response:

These sentences have been revised in light of feedback from all reviewers are, we hope, are more clear now.

**Lines 177-179:** It would be helpful to clarify why you determined these two databases were the predominant databases in the health sciences and social sciences, and, for example, why you chose to use Medline via Ovid instead of PubMed. I’m not passing judgment on your decision - only asking for clarification.

Author response:

Thank you for this! We added some language even earlier than this in order to clarify our decision to use the Ovid platform for these hedges, due to its increased functionality. We left in the existing language that explained the decision to use these two databases because of their size and popularity.

**Lines 187-189:** It would be helpful to state why you intentionally aligned with the [tiab] field. This is common for systematic review search methodology but it is good to explain why.

Author response:

Thank you for this suggestion. We have added an explanation in the relevant section about our use of the [tiab] field and we reproduce that language here:

We chose field codes that align with [tiab] in PubMed in the interest of capturing search terms in the title, abstract, or keyword fields of search results while reducing irrelevant results from additional metadata fields. Neither Ovid MEDLINE nor Ovid APA PsycInfo allow searching full text and other metadata fields (e.g., journal title and cited references) typically introduce unwanted noise. Searching title, abstract, controlled vocabulary terms, and author-provided keywords is standard practice in evidence synthesis searching.

**Lines 256-257:** Consider numbering the list for clarity. ... five categories: 1) Exclude, 2) No_Abstract, …

Author response:

Thanks for this suggestion. We have done so.

**Lines 280-284:** Consider moving the last two sentences in this paragraph describing how the articles were independently screened by two reviewers to the very beginning of the paragraph. Also, you state that screening was done by at least two “trained reviewers.” Are these trained reviewers the co-authors or other people?

Author response:

Thank you for this suggestion. We have revised this section significantly and trust that it will be simpler to read and less confusing.

**Figures 1 & 2:** You explain in your methods that the total number of articles included includes the included articles from the title and abstract screening combined with the included articles from the full-text screening. So, for figure 1, the total included articles is 1,324+339=1,663. This should be visualized in both figure 1 and 2.

**Lines 313-315:** It may be helpful to include a table with the precision calculations.

Author response:

Thank you for pointing this out. We have modified the figures to address this. We note also that the precision calculations in tables 2 & 3 are already indicated in their own columns.

**Tables 2 & 3:** Would be easier to read if abbreviations for INC, EXC, TA, and FT were spelled out on the table. Also, specifying OVID would be more accurate for both databases.

Author response:

We have left the tables with our abbreviations for the sake of space on the page and legibility. The cells would become too large if spelled out and still in the same font size; thus, we use abbreviations to be able to fit all seven columns in tables 2-5 and minimize the number of tables needed overall. In our view, it is pertinent to have the Ovid MEDLINE and Ovid APA PsycInfo results in the same tables and the disaggregated data visible in the same rows for ease of cross comparison for readers.

We did, however, specify Ovid everywhere relevant, including in tables.

**Lines 396-397:** May be helpful to specify the “other terms” that retrieve relevant articles for “transperson.”

Author response:

Thank you for raising this point. For the relevant article with the term “transperson*”, the term “transgender” also occurred in the title, abstract and MeSH heading term (Line 369-372 of the original submitted manuscript).

**Lines 464-469:** These two sentences about NLM thesaurus changes and the new nonbinary tag in PsycInfo can be further clarified to make it more explicit whether or not you were able to incorporate these changes into your search hedge. I don’t think you did because you completed the search hedge in 2024. This is okay, but the phrasing of the sentences could be more clear and explicit.’

Author response:

Thank you for pointing this out. Our manuscript specifies that the study validates searches as of June 2024 and that the appendix reports searches which incorporate thesaurus changes made after this date. Such changes are annotated in the supplementary information.

**Lines 476-478:** In final draft, include where (what platform or website) these continuously updated hedges will be made publicly available.

Author response:

As stated above in previous responses to Reviewer 1, we hope to maintain updates to these search hedges and disseminate them via platforms frequently utilized by expert searchers in the social sciences and the health sciences, such as The ISSG Search Filter Resource (<https://sites.google.com/a/york.ac.uk/issg-search-filters-resource/home>). This poses a small catch-22 for our author team because acceptance into repositories is contingent upon successful peer-reviewed publication, or conference presentation. Upon acceptance we will be enabled to share the hedges with the ISSG Search Filter Resource.
