# Supplementary material for: Development and validation of search hedges for Transgender and Gender Diverse (TGD) populations in Ovid MEDLINE and Ovid APA PsycInfo
Source: PLoS One. 2026 Jun 12;21(6):e0351303. doi: 10.1371/journal.pone.0351303 (PMC13262833; doi:10.1371/journal.pone.0351303)
Supplement: Peer review history

# Peer review: Decision Letter 0

**DOI:** 10.1371/journal.pone.0351303.r001

**Authors:** Christina Roberts

17 Mar 2026

PONE-D-26-08417

Development and validation of search hedges for transgender and gender diverse (TGD) populations in Ovid MEDLINE and Ovid APA PsycInfo

PLOS One

Dear Dr. Everhart,

Thank you for submitting your manuscript to PLOS ONE. After careful consideration, we feel that it has merit but does not fully meet PLOS ONE’s publication criteria as it currently stands. Therefore, we invite you to submit a revised version of the manuscript that addresses the points raised during the review process.

Please submit your revised manuscript by May 01 2026 11:59PM. If you will need more time than this to complete your revisions, please reply to this message or contact the journal office at plosone@plos.org. When you're ready to submit your revision, log on to https://www.editorialmanager.com/pone/ and select the 'Submissions Needing Revision' folder to locate your manuscript file.

Please include the following items when submitting your revised manuscript:

- A letter that responds to each point raised by the academic editor and reviewer(s). You should upload this letter as a separate file labeled 'Response to Reviewers'.
- A marked-up copy of your manuscript that highlights changes made to the original version. You should upload this as a separate file labeled 'Revised Manuscript with Track Changes'.
- An unmarked version of your revised paper without tracked changes. You should upload this as a separate file labeled 'Manuscript'.

If you would like to make changes to your financial disclosure, please include your updated statement in your cover letter. Guidelines for resubmitting your figure files are available below the reviewer comments at the end of this letter.

We look forward to receiving your revised manuscript.

Kind regards,

Christina M. Roberts, M.D., M.P.H.

Academic Editor

PLOS One

**Journal Requirements:**

1. When submitting your revision, we need you to address these additional requirements.

Please ensure that your manuscript meets PLOS ONE's style requirements, including those for file naming. The PLOS ONE style templates can be found at

https://journals.plos.org/plosone/s/file?id=wjVg/PLOSOne_formatting_sample_main_body.pdf and

https://journals.plos.org/plosone/s/file?id=ba62/PLOSOne_formatting_sample_title_authors_affiliations.pdf

2. Thank you for stating the following in the Acknowledgments Section of your manuscript:

The authors would like to thank Meng Wang (University of Calgary) for consultation on statistical analysis. This project received no direct grant from any funding agency in the public, commercial, or not-for-profit sectors.

We note that you have provided funding information that is not currently declared in your Funding Statement. However, funding information should not appear in the Acknowledgments section or other areas of your manuscript. We will only publish funding information present in the Funding Statement section of the online submission form.

Please remove any funding-related text from the manuscript and let us know how you would like to update your Funding Statement. Currently, your Funding Statement reads as follows:

The author(s) received no specific funding for this work.

Please include your amended statements within your cover letter; we will change the online submission form on your behalf.

3. When completing the data availability statement of the submission form, you indicated that you will make your data available on acceptance. We strongly recommend all authors decide on a data sharing plan before acceptance, as the process can be lengthy and hold up publication timelines. Please note that, though access restrictions are acceptable now, your entire data will need to be made freely accessible if your manuscript is accepted for publication. This policy applies to all data except where public deposition would breach compliance with the protocol approved by your research ethics board. If you are unable to adhere to our open data policy, please kindly revise your statement to explain your reasoning and we will seek the editor's input on an exemption. Please be assured that, once you have provided your new statement, the assessment of your exemption will not hold up the peer review process.

4. If the reviewer comments include a recommendation to cite specific previously published works, please review and evaluate these publications to determine whether they are relevant and should be cited. There is no requirement to cite these works unless the editor has indicated otherwise.

5. Please review your reference list to ensure that it is complete and correct. If you have cited papers that have been retracted, please include the rationale for doing so in the manuscript text, or remove these references and replace them with relevant current references. Any changes to the reference list should be mentioned in the rebuttal letter that accompanies your revised manuscript. If you need to cite a retracted article, indicate the article’s retracted status in the References list and also include a citation and full reference for the retraction notice.

**Additional Editor Comments:**

Thank you for submitting your manuscript to PLOS One. The reviewers and I feel your work has merit; however, additional revisions are needed before your manuscript is acceptable for publication. The reviewers have several requests for clarification that I would like you to address if you choose to revise and resubmit your manuscript. I have elaborated on some of their suggestions below and would like you to consider these clarifications when addressing the reviewer's suggestions.

Reviewer 1's suggestion:

"The manuscript would benefit from more explicit discussion of how the search hedges translate across databases. Because indexing systems, controlled vocabularies, and field structures vary substantially between databases, readers would benefit from guidance on:

* which elements of the hedge are database-specific,

* how subject headings were mapped across databases, and

* what adaptations are required for implementation in other search platforms."

Editor's elaboration

You touch on this issue in your manuscript, but provide minimal details:

Line 115: "Although these hedges are designed and validated on the Ovid platform, they are constructed in such a way to facilitate translation to other platforms, including PubMed, EBSCOhost, ProQuest, and APA PsycNet."

You can expand on this statement in your manuscript if you want. However, I am concerned that a review of how to adapt search hedges for use in different databases is beyond the scope of this manuscript. You can consider providing a reference to an article discussing this topic as an alternative to the detailed discussion suggested by Reviewer 1.

Reviewer 1's suggestion:

"When utilizing a search hedge, information professionals and researchers should consider if the hedge has been validated, if it optimizes sensitivity or precision, when it was last updated, and whether it needs to be adapted to suit their specific research question."

This is a useful recommendation, but it would benefit from contextualization. The authors might discuss how often validated search hedges exist in practice, in which fields they are most common (e.g., clinical medicine vs. social sciences), and for approximately how many types of search queries such hedges have been developed.

Editor's elaboration:

I feel Reviewer 1's suggestion has merit and your paper would benefit from additional contextualization. However, I am concerned that a review of search hedges use is beyond the scope of this paper. Consider directing readers to papers addressing this general information about search hedge use, such as reference 4, as an alternative to a full discussion.

Reviewer 1's suggestions:

"Librarian co-authors [REDACTED] and [REDACTED] will maintain and periodically update the hedge to reflect language, indexing practices, and community usage developments."

It would be helpful to specify where these updates will be maintained. The authors might consider discussing whether a platform could be used to host and version search hedges. For example, research infrastructures or methodological repositories could serve as dissemination platforms. In the digital humanities and social sciences, infrastructures such as the *SSH Marketplace* operated by *DARIAH-EU* publish methodological resources; a similar approach could potentially be used for search hedges.

"Through active maintenance and user adaptation, these hedges should be refined and expanded to reflect changes in language, indexing preferences, and the needs of researchers working with TGD populations over time."

The authors should clarify whose responsibility such maintenance would be, how updates might be coordinated, and how frequently revisions should occur.

Editor's Elaboration:

I agree that it is important to update search hedges to "to reflect language, indexing practices, and community usage developments" and to ensure dissemination of these search hedges for use by others. However, I feel your paper should focus on the work being presented rather than describing work you may do in the future. Consider limiting your discussion to the need for this work to occur in the future. You could also direct readers to any currently available dissemination platforms for search hedges via a reference, in the manuscript itself, or via a supplemental appendix.

Reviewer 3's suggestion:

"In your previous published protocol, your team acknowledged a possible conflict of interest with two authors of the protocol overlapping with Knowsy. This might need to be acknowledged in this manuscript as well."

Editor's elaboration: Please include this in your discussion

Reviewer 3's suggestion:

"It looks like Table 3 is listed in the manuscript before Table 2. Not sure if this matters for the journal formatting, but pointing it out just in case. It does refer to the correct table so is probably okay.

Editor's elaboration: Please number your tables in the order they appear in the manuscript.

Christina Roberts

[Note: HTML markup is below. Please do not edit.]

Reviewers' comments:

Reviewer's Responses to Questions

**Comments to the Author**

1. Is the manuscript technically sound, and do the data support the conclusions?

The manuscript must describe a technically sound piece of scientific research with data that supports the conclusions. Experiments must have been conducted rigorously, with appropriate controls, replication, and sample sizes. The conclusions must be drawn appropriately based on the data presented.

Reviewer #1: Partly

Reviewer #2: Yes

Reviewer #3: Yes

**********

2. Has the statistical analysis been performed appropriately and rigorously?

Reviewer #1: N/A

Reviewer #2: Yes

Reviewer #3: Yes

**********

3. Have the authors made all data underlying the findings in their manuscript fully available?

The PLOS Data policy requires authors to make all data underlying the findings described in their manuscript fully available without restriction, with rare exception (please refer to the Data Availability Statement in the manuscript PDF file). The data should be provided as part of the manuscript or its supporting information, or deposited to a public repository. For example, in addition to summary statistics, the data points behind means, medians and variance measures should be available. If there are restrictions on publicly sharing data—e.g. participant privacy or use of data from a third party—those must be specified.

Reviewer #1: No

Reviewer #2: Yes

Reviewer #3: Yes

**********

4. Is the manuscript presented in an intelligible fashion and written in standard English?

PLOS ONE does not copyedit accepted manuscripts, so the language in submitted articles must be clear, correct, and unambiguous. Any typographical or grammatical errors should be corrected at revision, so please note any specific errors here.

Reviewer #1: Yes

Reviewer #2: Yes

Reviewer #3: Yes

**********

5. Review Comments to the Author

Please use the space provided to explain your answers to the questions above. You may also include additional comments for the author, including concerns about dual publication, research ethics, or publication ethics. (Please upload your review as an attachment if it exceeds 20,000 characters)

**Reviewer #1:** This manuscript addresses an important methodological gap in evidence synthesis by developing and validating search hedges intended to improve retrieval of literature related to transgender and gender diverse (TGD) populations. The work is timely given the rapid evolution of terminology related to gender diversity and the well-documented challenges of constructing comprehensive search strategies in this area. The manuscript demonstrates a thoughtful attempt to balance methodological rigor with responsiveness to evolving language. However, several areas would benefit from clarification, expansion, and methodological justification.

The manuscript would benefit from greater clarity and consistency in terminology. The text alternates between several related but distinct terms such as “transgender and gender diverse”, “transgender”, “diverse and expansive gender identities”, and “2SLGBTQIA+”. These terms refer to overlapping but not identical populations and conceptual frameworks. The authors should define these terms clearly and justify their choice of terminology in relation to the scope of the search hedges.

Additionally, it should be acknowledged that some less advantaged communities do not prefer the term “minority”.

The manuscript refers to validation using sensitivity and precision, but it would be clearer to use the more established information-retrieval terminology of *recall* and *precision*. The authors note that:

"search hedges are validated by testing their sensitivity, also referred to as recall, against a 'gold standard' set of known relevant records."

A reference should be provided for this methodological approach, or the authors should verify whether a canonical reference (e.g., from information science or systematic review methodology literature) exists. The statement that hedges are evaluated by:

"their precision, which is the ratio of relevant to irrelevant records retrieved [6]. A challenge of search hedge development is balancing sensitivity and precision, as they are inversely related"

is correct but would benefit from clearer explanation of how this trade-off was operationalized in this study.

The manuscript also states that:

"The hedges were validated for sensitivity using a gold standard set of 144 articles from the Knowsy portal of evidence syntheses tagged as Two-Spirit, transgender, or gender non-binary."

However, further methodological detail is required. For example:

- How were the 144 articles selected and curated in the Knowsy portal?

- Why was this particular dataset chosen as the gold standard rather than other potential sources?

More detail is also needed regarding the screening process described in the manuscript:

"an international research team of subject experts independently screened a randomized sample of search results in a two-stage screening process with an additional screener resolving disputes."

The manuscript should specify how many reviewers were involved, how disagreements were resolved, and whether inter-rater agreement or another measure of consistency was assessed.

The authors should consider whether Homosaurus, a controlled vocabulary widely used for LGBTQ+ information organization, could have been used to identify relevant search terms. If it was considered but not used, the manuscript could explain why. Given that the study addresses evolving terminology, engagement with existing domain-specific vocabularies would strengthen the methodological justification.

The manuscript would benefit from more explicit discussion of how the search hedges translate across databases. Because indexing systems, controlled vocabularies, and field structures vary substantially between databases, readers would benefit from guidance on:

* which elements of the hedge are database-specific,

* how subject headings were mapped across databases, and

* what adaptations are required for implementation in other search platforms.

Some sentences require clarification or expansion. For example, the manuscript states:

"Social science journals have been slower to adopt structured abstracts which can be a barrier to efficient search development [7,8]."

In its current form, this sentence appears disconnected from the surrounding paragraph. The authors should either expand on how the absence of structured abstracts affects search hedge development or remove the sentence.

Similarly, the statement:

"Reviews on topics with developing or dynamic terminology, especially require a more sensitive strategy."

is unclear and should be elaborated. The authors could specify why dynamic terminology—such as evolving gender identity terms—creates challenges for search strategy construction and why higher recall may be necessary in such contexts.

The manuscript advises that:

"When utilizing a search hedge, information professionals and researchers should consider if the hedge has been validated, if it optimizes sensitivity or precision, when it was last updated, and whether it needs to be adapted to suit their specific research question."

This is a useful recommendation, but it would benefit from contextualization. The authors might discuss how often validated search hedges exist in practice, in which fields they are most common (e.g., clinical medicine vs. social sciences), and for approximately how many types of search queries such hedges have been developed.

The authors should also acknowledge limitations of relying on validation against a gold standard dataset. For example:

* new articles published after the creation of the gold standard may not be represented,

* the gold standard itself may omit relevant studies,

* validation results may therefore underestimate or overestimate actual recall.

The manuscript notes that:

"Existing search strategies often fail to capture the full range of gender diversity — particularly when it comes to culturally specific gender identities, Indigenous systems of sex and gender, and evolving gender terminology."

This is an important point but requires additional references and engagement with existing research on the discoverability of LGBTQ+ scholarship and culturally specific gender identities in bibliographic databases.

The authors also state that:

"while other terms that do not currently retrieve many records have been deliberately included for future growth as scholarly research adapts to emerging terminology"

The manuscript should clarify what “future growth” refers to and whose needs this anticipates (e.g., future researchers, evolving academic terminology, indexing updates). Providing concrete examples of such terms would improve clarity.

There are several stylistic issues that could be improved. For example, the sentence beginning in row 98 is unusually long and would benefit from being split or rewritten for clarity.

The manuscript states that:

"Librarian co-authors [REDACTED] and [REDACTED] will maintain and periodically update the hedge to reflect language, indexing practices, and community usage developments."

It would be helpful to specify where these updates will be maintained. The authors might consider discussing whether a platform could be used to host and version search hedges. For example, research infrastructures or methodological repositories could serve as dissemination platforms. In the digital humanities and social sciences, infrastructures such as the *SSH Marketplace* operated by *DARIAH-EU* publish methodological resources; a similar approach could potentially be used for search hedges.

Relatedly, the manuscript states:

"Through active maintenance and user adaptation, these hedges should be refined and expanded to reflect changes in language, indexing preferences, and the needs of researchers working with TGD populations over time."

The authors should clarify whose responsibility such maintenance would be, how updates might be coordinated, and how frequently revisions should occur.

Further methodological clarification is required regarding the screening process. In particular:

* Who conducted the full-text reviews?

* How many reviewers were involved?

* How was subjectivity addressed during relevance assessment?

For example, the manuscript notes that:

"the majority of the articles in this category were coded as TGD-relevant upon examination of the full-text"

The criteria used to determine relevance should be clearly defined, and the procedures for resolving disagreements should be described.

Overall, the manuscript addresses an important methodological challenge and has the potential to make a valuable contribution to evidence synthesis and information retrieval in gender diversity research. Strengthening the methodological transparency, clarifying terminology, and expanding discussion of validation and sustainability would substantially improve the manuscript.

**Reviewer #2:** I thoroughly enjoyed reading this paper and thought it was well researched and a much needed addition to publications on this topic. I have a few suggested edits, which are:

-Lines 77-78: I found the sentence structure difficult here. Consider changing to "Reviews on topics with developing or dynamic terminology require a more sensitive strategy."

-Lines 79-80: Suggest adding consideration of which database platform the hedge is for

-Line 93: remove the "s" from the end of 'retrieves' as it is referring to 'search hedges'

-Line 145: Consider defining the term "cisgender" when it first appears

-Line 449: Remove "At the same time" from beginning of sentence as it is the beginning of a new section and reads as a continuation of a paragraph instead.

**Reviewer #3:** Overall, this is a very well written, organized, and clear manuscript on a very relevant topic that fills a gap in research. I believe the methods and conclusions are sound. My suggestions focus on ways to improve clarity.

Lines 74-76: The terms precision and sensitivity can be further defined to provide clarity. I recommend incorporating a brief summary of your definitions of these terms in your previously published protocol.

Lines 96-98: The term “excessive noise” should be defined in terms of what it actually means. In this context, you are using “excessive noise” as a metaphor and not everyone reading this will understand what you mean. You use similar terms (e.g., noisy) throughout the manuscript. Either define it the first time you use it so that it is clear what you mean, or use more precise non-metaphorical descriptions.

Line 115: This is the first time you mention Medline and PsycInfo in the main text (other than the title and abstract). Include Ovid here like you do in the title and abstract just to make it clear that this is the platform you used.

Line 118: Methods section should have a subheading for the Gold Standard Set. The gold standard set--what it includes, how it was developed, why it was selected, and why it is appropriate--are all very important to the validation methods and resulting outcomes. This should be described in detail. Would also be good to briefly discuss external validation vs internal as you did in the protocol.

Line 135: In the “search term selection” section, you state that “we consulted the initial searches used to populate Knowsy,” but I don’t see that this list is publicly available on the Knowsy website, but I may have missed it. Please specify if you got the list from the public website or if you got the list from one of the co-authors in this manuscript who helped to develop or continue to maintain Knowsy. In your previous published protocol, your team acknowledged a possible conflict of interest with two authors of the protocol overlapping with Knowsy. This might need to be acknowledged in this manuscript as well.

Lines 140-143: This last sentence (starting with “cultural specific terms” is a little confusing and could be revised into more simple or straightforward terms for clarity.

Lines 177-179: It would be helpful to clarify why you determined these two databases were the predominant databases in the health sciences and social sciences, and, for example, why you chose to use Medline via Ovid instead of PubMed. I’m not passing judgment on your decision - only asking for clarification.

Lines 187-189: It would be helpful to state why you intentionally aligned with the [tiab] field. This is common for systematic review search methodology but it is good to explain why.

Lines 256-257: Consider numbering the list for clarity. ... five categories: 1) Exclude, 2) No_Abstract, …

Lines 280-284: Consider moving the last two sentences in this paragraph describing how the articles were independently screened by two reviewers to the very beginning of the paragraph. Also, you state that screening was done by at least two “trained reviewers.” Are these trained reviewers the co-authors or other people?

Line 291: It looks like Table 3 is listed in the manuscript before Table 2. Not sure if this matters for the journal formatting, but pointing it out just in case. It does refer to the correct table so is probably okay.

Figures 1 & 2: You explain in your methods that the total number of articles included includes the included articles from the title and abstract screening combined with the included articles from the full-text screening. So, for figure 1, the total included articles is 1,324+339=1,663. This should be visualized in both figure 1 and 2.

Lines 313-315: It may be helpful to include a table with the precision calculations.

Tables 2 & 3: Would be easier to read if abbreviations for INC, EXC, TA, and FT were spelled out on the table. Also, specifying OVID would be more accurate for both databases.

Line 377: Again, specify OVID when naming the databases.

Lines 396-397: May be helpful to specify the “other terms” that retrieve relevant articles for “transperson.”

Lines 464-469: These two sentences about NLM thesaurus changes and the new nonbinary tag in PsycInfo can be further clarified to make it more explicit whether or not you were able to incorporate these changes into your search hedge. I don’t think you did because you completed the search hedge in 2024. This is okay, but the phrasing of the sentences could be more clear and explicit.

Lines 476-478: In final draft, include where (what platform or website) these continuously updated hedges will be made publicly available.

Additional Limitations: Your previously published protocol provides more information about the Knowsy gold standard set of articles, which were collected in 2019. This should be acknowledged as a limitation because it means that your gold standard set does not include recent articles published between 2019-2026, and that your search hedge reflects this. This can be connected back to your discussion regarding the need to consistently update due to evolving language and database functionality and goal of continuing to update and maintain this hedge.

**********

6. PLOS authors have the option to publish the peer review history of their article (what does this mean?). If published, this will include your full peer review and any attached files.

If you choose “no”, your identity will remain anonymous but your review may still be made public.

**Do you want your identity to be public for this peer review?** For information about this choice, including consent withdrawal, please see our Privacy Policy.

Reviewer #1: No

Reviewer #2: **Yes:** Brooke Ballantyne Scott

Reviewer #3: **Yes:** Hannah M. Schilperoort

**********

[NOTE: If reviewer comments were submitted as an attachment file, they will be attached to this email and accessible via the submission site. Please log into your account, locate the manuscript record, and check for the action link "View Attachments". If this link does not appear, there are no attachment files.]

To ensure your figures meet our technical requirements, please review our figure guidelines: https://journals.plos.org/plosone/s/figures

You may also use PLOS’s free figure tool, NAAS, to help you prepare publication quality figures: https://journals.plos.org/plosone/s/figures#loc-tools-for-figure-preparation.

NAAS will assess whether your figures meet our technical requirements by comparing each figure against our figure specifications.

# Author comment: Author response to Decision Letter 1

**DOI:** 10.1371/journal.pone.0351303.r002

5 May 2026

Reviewer 1:

The manuscript would benefit from more explicit discussion of how the search hedges translate across databases. Because indexing systems, controlled vocabularies, and field structures vary substantially between databases, readers would benefit from guidance on:

* which elements of the hedge are database-specific,

* how subject headings were mapped across databases, and

* what adaptations are required for implementation in other search platforms.

Editor's elaboration:

You touch on this issue in your manuscript, but provide minimal details:

Line 115: "Although these hedges are designed and validated on the Ovid platform, they are constructed in such a way to facilitate translation to other platforms, including PubMed, EBSCOhost, ProQuest, and APA PsycNet."

You can expand on this statement in your manuscript if you want. However, I am concerned that a review of how to adapt search hedges for use in different databases is beyond the scope of this manuscript. You can consider providing a reference to an article discussing this topic as an alternative to the detailed discussion suggested by Reviewer 1.

Author response:

The following text has been added to the first paragraph of subsection “Search hedge structure” under the Methods section and appears beginning at line 179 in the manuscript.

Search hedges can be translated across different databases and between different interfaces and platforms that search the same database. Glanville et al. (2019) provide a case study detailing how to translate a validated search hedge from one platform to another.

For ease of translation across different databases, the controlled vocabulary terms for each search hedge have been grouped in the first line of each respective hedge. These database-specific controlled vocabulary terms are the MeSH terms for Ovid MEDLINE and the APA Thesaurus of Psychological Index terms for Ovid APA PsycInfo. The other database-specific elements that would need to be translated for other databases or platforms would be the field codes (such as ".ti,ab,kf,kw,ot,oa,cl." or ".tw.") and the adjacency operator ("adj5") that we tried to minimize.The human limiter for Ovid MEDLINE (line 12) is specific to that database to mitigate indexing errors. We tried to limit the need to translate other database-specific syntax such as advanced wildcard operators to reduce potential errors in translation. In addition, we address this in the limitations section in the original manuscript and have kept it in this revision.

Line 63: "When utilizing a search hedge, information professionals and researchers should consider if the hedge has been validated, if it optimizes sensitivity or precision, when it was last updated, and whether it needs to be adapted to suit their specific research question."

This is a useful recommendation, but it would benefit from contextualization. The authors might discuss how often validated search hedges exist in practice, in which fields they are most common (e.g., clinical medicine vs. social sciences), and for approximately how many types of search queries such hedges have been developed.

Editor's elaboration:

I feel Reviewer 1's suggestion has merit and your paper would benefit from additional contextualization. However, I am concerned that a review of search hedges use is beyond the scope of this paper. Consider directing readers to papers addressing this general information about search hedge use, such as reference 4, as an alternative to a full discussion.

Author Response:

We agree that some more context about utilizing search hedges would be beneficial to our paper, although we share the editor's point that some of the points Reviewer 1 brought up are outside the scope of this paper.

To address this concern we revised the introduction section in several places. First, our second paragraph now contains further information about the utility of search hedges, generally (lines 62-63 and 65-69). Second, we expanded upon the paragraph this reviewer cites beginning just after the sentence they quote (lines 87-). The text for each of these revisions appears below:

62-63 “...as pre-constructed components of a larger, comprehensive search strategy in evidence synthesis reports…”

Lines 65-69 “In particular, search hedges can improve the comprehensiveness and sensitivity of database queries when searching for complex concepts and populations that have significant language variability. Researchers can incorporate search hedges as an integral part of their approach to evidence synthesis, thus making their searches more complete and reducing the risk of missing relevant literature.”

Lines 87-92 “While a more thorough explanation of search hedges as tools for more general use is beyond the scope of this study, others have described the landscape of existing topic search hedges. There are a variety of sources for validated search hedges. For example, the InterTASC Information Specialists’ Sub-Group Search Filter Resource provides access to published and unpublished search hedges as well as information about how to critically develop and critically appraise search hedges.

Line 442: "Librarian co-authors [REDACTED] and [REDACTED] will maintain and periodically update the hedge to reflect language, indexing practices, and community usage developments."

It would be helpful to specify where these updates will be maintained. The authors might consider discussing whether a platform could be used to host and version search hedges. For example, research infrastructures or methodological repositories could serve as dissemination platforms. In the digital humanities and social sciences, infrastructures such as the *SSH Marketplace* operated by *DARIAH-EU* publish methodological resources; a similar approach could potentially be used for search hedges

Line 89: "Through active maintenance and user adaptation, these hedges should be refined and expanded to reflect changes in language, indexing preferences, and the needs of researchers working with TGD populations over time."

The authors should clarify whose responsibility such maintenance would be, how updates might be coordinated, and how frequently revisions should occur.

Editor's Elaboration:

I agree that it is important to update search hedges to "to reflect language, indexing practices, and community usage developments" and to ensure dissemination of these search hedges for use by others. However, I feel your paper should focus on the work being presented rather than describing work you may do in the future. Consider limiting your discussion to the need for this work to occur in the future. You could also direct readers to any currently available dissemination platforms for search hedges via a reference, in the manuscript itself, or via a supplemental appendix.

Author response:

Thank you for the suggestion to add information about search hedge dissemination platforms. See our comment above for the language we added that directs readers to an example of a repository for existing search filters. As discussed by the editor, we wanted to focus on the presentation of our current work rather than describing potential future work. We also added to the final sentence of the manuscript, which this reviewer cites, to name that ongoing updates are part of our dissemination plan.

The manuscript would benefit from greater clarity and consistency in terminology. The text alternates between several related but distinct terms such as “transgender and gender diverse”, “transgender”, “diverse and expansive gender identities”, and “2SLGBTQIA+”. These terms refer to overlapping but not identical populations and conceptual frameworks. The authors should define these terms clearly and justify their choice of terminology in relation to the scope of the search hedges.

Additionally, it should be acknowledged that some less advantaged communities do not prefer the term “minority”.

Author response:

Throughout the article, we refer to the population the hedges are about as "transgender and gender diverse" wherever possible to acknowledge that the scope of our search hedges are designed to capture transgender people in addition to gender diverse terminology for people who may or may not consider themselves transgender, but who do not identify as their assigned sex at birth.

When we refer to "2SLGBTQIA+," we are discussing the larger spectrum of sexuality and gender identity which provides unique challenges for identifying literature on transgender and gender diverse populations.

We acknowledge that many of the terms used throughout the search hedges and in this article may be antiquated or offensive. Searches supporting evidence syntheses require robust and comprehensive searches to identify all potentially relevant literature. We have also revised the few places where we used the minority language ourself to either clarify our intention, i.e., discussing specific results, or to change it to our TGD overarching terminology.

The manuscript refers to validation using sensitivity and precision, but it would be clearer to use the more established information-retrieval terminology of *recall* and *precision*. The authors note that: "search hedges are validated by testing their sensitivity, also referred to as recall, against a 'gold standard' set of known relevant records."

A reference should be provided for this methodological approach, or the authors should verify whether a canonical reference (e.g., from information science or systematic review methodology literature) exists. The statement that hedges are evaluated by: "their precision, which is the ratio of relevant to irrelevant records retrieved [6]. A challenge of search hedge development is balancing sensitivity and precision, as they are inversely related" is correct but would benefit from clearer explanation of how this trade-off was operationalized in this study.

Author response:

We initially name sensitivity on line 76 in the original manuscript and in the same sentence state that it is also referred to as recall. We’ve now added citations [6,7] to this sentence to indicate our decision to use sensitivity is consistent with Campbell and Cochrane guidance.

"The hedges were validated for sensitivity using a gold standard set of 144 articles from the Knowsy portal of evidence syntheses tagged as Two-Spirit, transgender, or gender non-binary."

However, further methodological detail is required. For example:

- How were the 144 articles selected and curated in the Knowsy portal?

- Why was this particular dataset chosen as the gold standard rather than other potential sources?

The authors should also acknowledge limitations of relying on validation against a gold standard dataset. For example:

* new articles published after the creation of the gold standard may not be represented,

* the gold standard itself may omit relevant studies,

* validation results may therefore underestimate or overestimate actual recall.

Author response:

We appreciate the reviewer’s feedback and concerns. And while the methodological choice to rely on Knowsy as a gold standard set reflects upon this article, the methods behind that data set are not part of this study. In addition, there are no alternatives that our author team are aware of that are comprehensive and not delimited by other hedge portions (such as a full PICO). Below we address these concerns as they pertain to the study at hand.

There are multiple approaches to identifying a gold standard dataset, predominantly hand searching and relative recall, which we addressed in lines 78-80 of the original manuscript and have left in the revision. We have now added a citation to that sentence that addresses the use of gold standard sets for validation. Before addressing the specifics of our hedge and the gold standard set we used to validate it, we want to also address this reviewer’s bulleted comments about limitations. These are true of all gold standard sets (temporal limits, omission, under/overestimation). While our measure of sensitivity is based largely on Knowsy, we validated a large random sample of all records returned from our hedge in both databases. The existing explanation in our methods of that process, in our view, demonstrates that our methods for validation did not rely solely on a gold standard set, but rather on a robust process for screening and review.

The Knowsy dataset was created from searches of MEDLINE, APA PsycInfo, Embase, and CINAHL.designed by a librarian not involved in this study. The Knowsy team manually screened records and assigned population tags. Thus, we were able to utilize an existing dataset created independently from the librarians who developed these search hedges. For the current gold standard dataset, we used all studies tagged with the terms gender identity, Two-Spirit, transgender, or gender non-binary.

Moreover, for this specific hedge, which is focused on TGD populations, there is good reason to use an ‘older’ dataset as a gold standard. Because of the lag between the introduction of new terminology that TGD communities actually use and publication that deploys that terminology in the lieterature, we would be more concerned with sensitivity for a dataset that is older. This is because we would want to make sure that our hedge is as comprehensive and temporally unbiased as possible. This also led us to leave in many of the terms that didn’t necessarily have any unique hits so that it can be an evolving search hedge and capture new things as they are being published. On the other end of the spectrum, sensitivity to a gold standard, in our case Knowsy, that included all literature up to 2014 would indicate that our hedge performs well with historical data as well, which makes it more useful to a wider array of researchers and scholars, including those working in history and related fields who may be more concerned with a larger temporal period than just the most up to date evidence available.

More detail is also needed regarding the screening process described in the manuscript:

"an international research team of subject experts independently screened a randomized sample of search results in a two-stage screening process with an additional screener resolving disputes."

The manuscript should specify how many reviewers were involved, how disagreements were resolved, and whether inter-rater agreement or another measure of consistency was assessed.

…"the majority of the articles in this category were coded as TGD-relevant upon examination of the full-text" The criteria used to determine relevance should be clearly defined, and the procedures for resolving disagreements should be described.

Author response:

Members of the author team for this study have already published a protocol paper on our methods, which we cite. While we recognize that this article under consideration should stand on its own, we do consider some of the details to be out of scope. In that protocol paper we outline the screening process thoroughly and share only the most immediately relevant details here, which is not an uncommon practice in scientific literature. We have, however, included a interrater reliability measure at the reviewer’s suggestion in hopes that it clarifies the effectiveness of our approach. And, where relevant, we have added further language to explain the inclusion criteria, while still directing readers to the protocol paper should they be especially interested in the details of our screening process.

More specifically, we have added further elaboration in the final paragraph of the “Validation” section of the Methods. In addition, we added some further language to clarify what constituted grounds for inclusion as TGD relevant. Finally, we have reported on agreement rates during both phases of screening to indicate how frequently reconciliation by consensus among study team members was necessary to resolve conflicts in screening records. There was a slight improvement in the full text phase from the title and abstract phase.

The authors should consider whether Homosaurus, a controlled vocabulary widely used for LGBTQ+ information organization, could have been used to identify relevant search terms. If it was considered but not used, the manuscript could explain why. Given that the

Attachment Submitted filename: response_to_reviewers.docx

# Peer review: Decision Letter 1

**DOI:** 10.1371/journal.pone.0351303.r003

**Authors:** Christina Roberts

26 May 2026

Development and validation of search hedges for transgender and gender diverse (TGD) populations in Ovid MEDLINE and Ovid APA PsycInfo

PONE-D-26-08417R1

Dear Dr. Everhart,

We’re pleased to inform you that your manuscript has been judged scientifically suitable for publication and will be formally accepted for publication once it meets all outstanding technical requirements.

Within one week, you’ll receive an e-mail detailing the required amendments. When these have been addressed, you’ll receive a formal acceptance letter and your manuscript will be scheduled for publication.

An invoice will be generated when your article is formally accepted. Please note, if your institution has a publishing partnership with PLOS and your article meets the relevant criteria, all or part of your publication costs will be covered. Please make sure your user information is up-to-date by logging into Editorial Manager at Editorial Manager® and clicking the ‘Update My Information' link at the top of the page. For questions related to billing, please contact billing support.

If your institution or institutions have a press office, please notify them about your upcoming paper to help maximize its impact. If they’ll be preparing press materials, please inform our press team as soon as possible -- no later than 48 hours after receiving the formal acceptance. Your manuscript will remain under strict press embargo until 2 pm Eastern Time on the date of publication. For more information, please contact onepress@plos.org.

Kind regards,

Christina M. Roberts, M.D., M.P.H.

Academic Editor

PLOS One

Additional Editor Comments (optional):

Reviewers' comments:

Reviewer's Responses to Questions

**Comments to the Author**

1. If the authors have adequately addressed your comments raised in a previous round of review and you feel that this manuscript is now acceptable for publication, you may indicate that here to bypass the “Comments to the Author” section, enter your conflict of interest statement in the “Confidential to Editor” section, and submit your "Accept" recommendation.

Reviewer #1: All comments have been addressed

Reviewer #2: All comments have been addressed

Reviewer #3: (No Response)

**********

2. Is the manuscript technically sound, and do the data support the conclusions?

The manuscript must describe a technically sound piece of scientific research with data that supports the conclusions. Experiments must have been conducted rigorously, with appropriate controls, replication, and sample sizes. The conclusions must be drawn appropriately based on the data presented.

Reviewer #1: (No Response)

Reviewer #2: Yes

Reviewer #3: Yes

**********

3. Has the statistical analysis been performed appropriately and rigorously?

Reviewer #1: (No Response)

Reviewer #2: Yes

Reviewer #3: Yes

**********

4. Have the authors made all data underlying the findings in their manuscript fully available?

The PLOS Data policy requires authors to make all data underlying the findings described in their manuscript fully available without restriction, with rare exception (please refer to the Data Availability Statement in the manuscript PDF file). The data should be provided as part of the manuscript or its supporting information, or deposited to a public repository. For example, in addition to summary statistics, the data points behind means, medians and variance measures should be available. If there are restrictions on publicly sharing data—e.g. participant privacy or use of data from a third party—those must be specified.

Reviewer #1: (No Response)

Reviewer #2: Yes

Reviewer #3: Yes

**********

5. Is the manuscript presented in an intelligible fashion and written in standard English?

PLOS ONE does not copyedit accepted manuscripts, so the language in submitted articles must be clear, correct, and unambiguous. Any typographical or grammatical errors should be corrected at revision, so please note any specific errors here.

Reviewer #1: (No Response)

Reviewer #2: Yes

Reviewer #3: Yes

**********

6. Review Comments to the Author

Please use the space provided to explain your answers to the questions above. You may also include additional comments for the author, including concerns about dual publication, research ethics, or publication ethics. (Please upload your review as an attachment if it exceeds 20,000 characters)

Reviewer #1: (No Response)

Reviewer #2: (No Response)

Reviewer #3: Thank you for your detailed responses to my comments and for your revisions. I believe this is a strong and relevant article that will add to the knowledge on TGD and LGBTQ+ health research.

I have two additional, connected suggestions.

Comment #1

I understand your hesitance in including a Gold Standard section in your Methods section given the reasons that you provided in your comments to both reviewers. However, both reviewers brought up similar concerns, which means that other readers may also have similar thoughts, which is why I think it would be beneficial to incorporate this into your article. This will give you more control of how readers interpret your work/article.

I continue to advocate that you include a Gold Standard section in your Methods, either as its own subheading or incorporated in the part of the broader Methods section that you think is the most appropriate. You do not need to go over the creation of the gold standard set in detail. Rather, provide a brief explanation of why you chose to use the Knowsy set as your gold standard instead of another set and why it was appropriate to do so. I am not passing judgment on your decision at all, but encourage you to briefly explain your choice in 1-3 sentences.

Comment #2

Thank you for adding the potential conflict of interest to the Limitations section. At the end of the new paragraph you state: “However, this is true for any gold standard data set.” This is true but good to acknowledge, especially for readers who are not as familiar with search hedge validation. My suggestion is to add one or two citations to support this last sentence.

**********

7. PLOS authors have the option to publish the peer review history of their article (what does this mean?). If published, this will include your full peer review and any attached files.

If you choose “no”, your identity will remain anonymous but your review may still be made public.

**Do you want your identity to be public for this peer review?** For information about this choice, including consent withdrawal, please see our Privacy Policy.

Reviewer #1: No

Reviewer #2: **Yes:** Brooke Ballantyne Scott

Reviewer #3: No

**********

# Editor report: Acceptance letter

**DOI:** 10.1371/journal.pone.0351303.r004

**Authors:** Christina Roberts

PONE-D-26-08417R1

PLOS One

Dear Dr. Everhart,

I'm pleased to inform you that your manuscript has been deemed suitable for publication in PLOS One. Congratulations! Your manuscript is now being handed over to our production team.

At this stage, our production department will prepare your paper for publication. This includes ensuring the following:

* All references, tables, and figures are properly cited

* All relevant supporting information is included in the manuscript submission,

* There are no issues that prevent the paper from being properly typeset

You will receive further instructions from the production team, including instructions on how to review your proof when it is ready. Please keep in mind that we are working through a large volume of accepted articles, so please give us a few days to review your paper and let you know the next and final steps.

Lastly, if your institution or institutions have a press office, please let them know about your upcoming paper now to help maximize its impact. If they'll be preparing press materials, please inform our press team within the next 48 hours. Your manuscript will remain under strict press embargo until 2 pm Eastern Time on the date of publication. For more information, please contact onepress@plos.org.

You will receive an invoice from PLOS for your publication fee after your manuscript has reached the completed accept phase. If you receive an email requesting payment before acceptance or for any other service, this may be a phishing scheme. Learn how to identify phishing emails and protect your accounts at https://explore.plos.org/phishing.

If we can help with anything else, please email us at customercare@plos.org.

Thank you for submitting your work to PLOS ONE and supporting open access.

Kind regards,

PLOS ONE Editorial Office Staff

on behalf of

Dr. Christina M. Roberts

Academic Editor

PLOS One
